# Latitude‐specific urbanization effects on life history traits in the damselfly *Ischnura elegans*

**DOI:** 10.1111/eva.13583

**Published:** 2023-08-01

**Authors:** Gemma Palomar, Guillaume Wos, Robby Stoks, Szymon Sniegula

**Affiliations:** ^1^ Institute of Nature Conservation Polish Academy of Sciences Krakow Poland; ^2^ Department of Genetics, Physiology, and Microbiology Complutense University of Madrid Madrid Spain; ^3^ Laboratory of Evolutionary Stress Ecology and Ecotoxicology KU Leuven Leuven Belgium

**Keywords:** global warming, invasive alien species, *Ischnura elegans*, pace‐of‐life syndrome, phenotypic plasticity, urbanization

## Abstract

Many species are currently adapting to cities at different latitudes. Adaptation to urbanization may require eco‐evolutionary changes in response to temperature and invasive species that may differ between latitudes. Here, we studied single and combined effects of increased temperatures and an invasive alien predator on the phenotypic response of replicated urban and rural populations of the damselfly *Ischnura elegans* and contrasted these between central and high latitudes. Adult females were collected in rural and urban ponds at central and high latitudes. Their larvae were exposed to temperature treatments (current [20°C], mild warming [24°C], and heat wave [28°C; for high latitude only]) crossed with the presence or absence of chemical cues released by the spiny‐cheek crayfish (*Faxonius limosus*), only present at the central latitude. We measured treatment effects on larval development time, mass, and growth rate. Urbanization type affected all life history traits, yet these responses were often dependent on latitude, temperature, and sex. Mild warming decreased mass in rural and increased growth rate in urban populations. The effects of urbanization type on mass were latitude‐dependent, with central‐latitude populations having a greater phenotypic difference. Urbanization type effects were sex‐specific with urban males being lighter and having a lower growth rate than rural males. At the current temperature and mild warming, the predator cue reduced the growth rate, and this independently of urbanization type and latitude of origin. This pattern was reversed during a heat wave in high‐latitude damselflies. Our results highlight the context‐dependency of evolutionary and plastic responses to urbanization, and caution for generalizing how populations respond to cities based on populations at a single latitude.

## 1 INTRODUCTION

Urbanization has emerged as a strong and widespread source of selection affecting plant and animal communities (Alberti, Correa, et al., 2017; Alberti, Marzluff, & Hunt, 2017; Catullo et al., 2019; Diamond et al., 2023). Urbanization is closely related to drivers of eco‐evolutionary change such as temperature and invasive species. Within the cities, the dense concentration of pavement, buildings, and other surfaces that absorb and retain heat creates ‘urban heat islands’ in which temperature is higher (Tam et al., 2015). Urbanization may also favour the introduction of invasive species reducing diversity by competitive exclusion of native species (McKinney, 2006). These phenomena together with other factors related to urbanization, such as pollution or noise, create particular environments within the cities that drive evolutionary changes in organisms, e.g. at the phenotypic and physiological levels, and in their interactions with other organisms (Alberti, Correa, et al., 2017; Alberti, Marzluff, & Hunt, 2017; Kotze et al., 2022; Lambert et al., 2021).

Global warming and latitudinal gradient might further interact with urbanization and alter organism's thermal plasticity and evolution (Diamond et al., 2023; Verheyen et al., 2019). These plastic and evolutionary responses may differ across the species latitudinal distribution because populations from different latitudes encounter different seasonal time constraints (Stoks et al., 2012). Since plastic responses are considered the first response to buffer the effects of novel environmental stressors (Fox et al., 2019), investigating reaction norms of organisms from urban and rural populations at different latitudes in response to warming and stress caused by invasive alien predators may provide valuable insights to forecast species' phenotypic response to the rapid local and global changes (Verheyen et al., 2019). Life history traits such as body size are responsive to urbanization, although the trends are taxon dependent. While a reduction in body size was observed in urban bumblebees (Eggenberger et al., 2019) and birds (Meillère et al., 2015), the opposite pattern was found in moths (Merckx, Kaiser, & Van Dyck, 2018). Urbanization effects may also be sex‐specific, e.g. urban males are bigger in butterflies (Kaiser et al., 2016). Available theories predict that when facing different ecological conditions, as generally observed along an urbanization or latitudinal gradient, organisms may be ranked from a slow‐ to fast‐living continuum (‘pace‐of‐life’ syndrome; Brans & De Meester, 2018; Réale et al., 2010). This may be reflected by a shift towards a faster pace‐of‐life with accelerated growth, rapid development, and lower body mass as urbanization increases (Brans & De Meester, 2018; Debecker et al., 2016). Therefore, as urbanization is expected to affect life history strategies and patterns of covariation between traits, it is important to consider multiple life history traits, both sexes and other interacting drivers of evolutionary change such latitude‐specific growth season length and warming, to better reflect anthropogenic impacts of cities on organisms.

In this study, we tested and contrasted the response of life history traits to single and combined thermal and invasive alien predator treatments between urban and rural populations from different latitudes. Special attention went to whether these life history responses were sex specific. We ran a common garden experiment on the damselfly *Ischnura elegans* from replicated rural and urban populations at high‐ (southern Sweden) and central‐ (southern Poland) latitudes. Larvae from field‐collected females were raised under one of three thermal regimes: current temperature (20°C), mild warming (24°C), and heat wave (28°C; for high‐ latitude populations only). In damselflies, urban populations were shown to grow slower than rural populations at central latitudes (Tüzün & Stoks, 2021), but whether such pattern is consistent across the species latitudinal range requires further investigation. At the central latitude, the higher annual temperatures and associated longer growth season allow more generations per year, but less time per generation, thereby causing higher seasonal time constraints (Corbet et al., 2006; Stoks et al., 2012). As the effect of urbanization on life history may differ between mild warming and heat wave temperatures, we tested both in the same study. The thermal treatment was crossed with a treatment where we manipulated the presence of chemical predator cues from an invasive alien predator, the spiny‐cheek crayfish, *Faxonius limosus*, which has been co‐occurring with Polish *I. elegans* populations for several decades, but has not yet been reported in Sweden. The spread of invasive crayfish predator has been largely mediated by human activities and urban areas represent an attractive environment for this species (Reynolds & Souty‐Grosset, 2011).

## 2 MATERIALS AND METHODS

### 2.1 Study species

*Ischnura elegans* is a common damselfly in Europe (Dijkstra & Schröter, 2020). Across a latitudinal gradient, populations are characterized by different number of generations per year (voltinism). At central latitudes (including Poland), populations are generally uni‐ and bivoltine, meaning one or two generations per year, respectively (Corbet et al., 2006; Norling, 2021). At high latitudes (including Sweden), populations are usually uni‐ and semivoltine, meaning that 1 or 2 years are required for completing one generation, respectively.

### 2.2 Study populations

**FIGURE 1:**
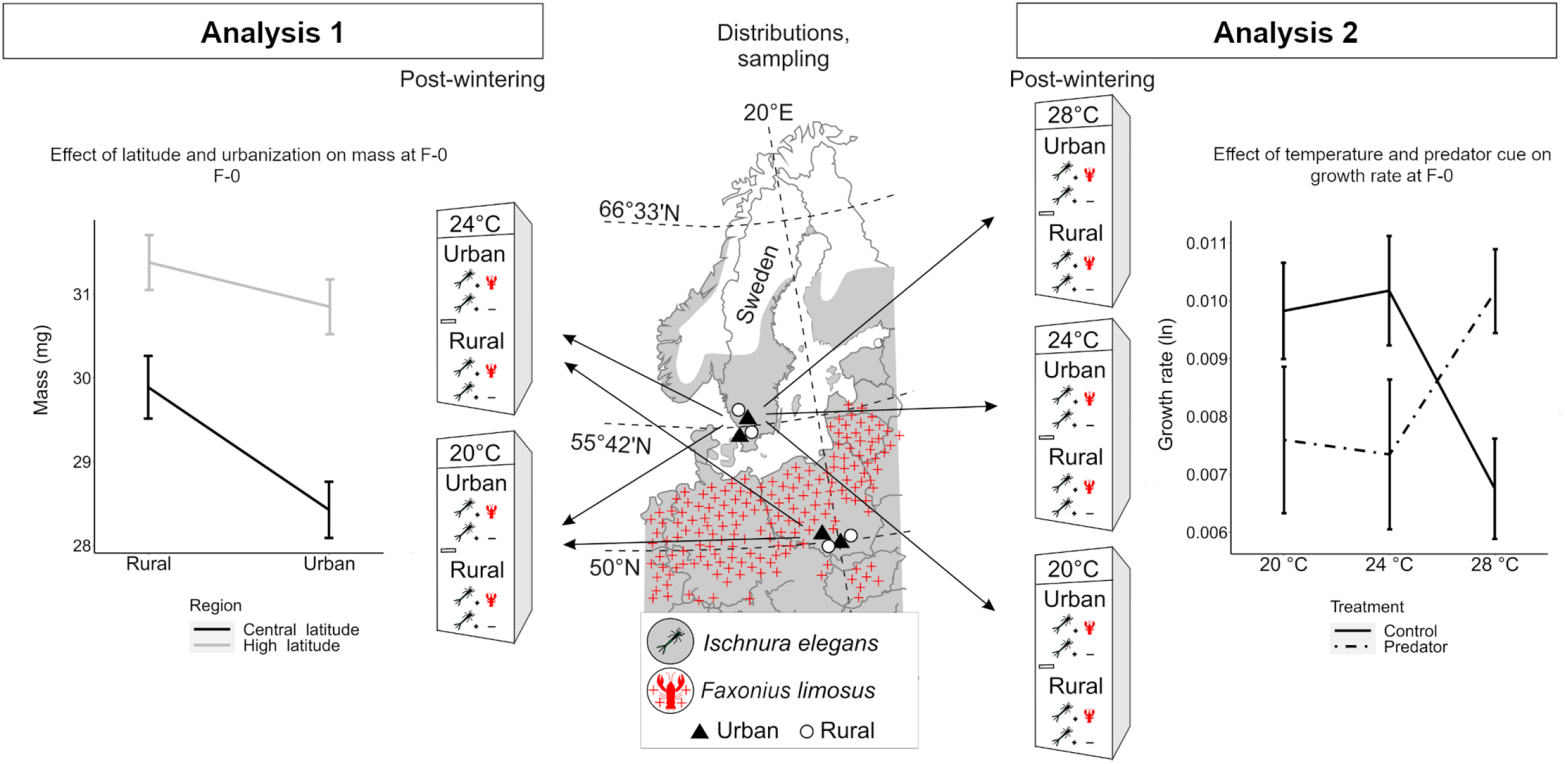
Summary of the experimental design with a map of our sampled populations in southern Sweden (high latitude) and southern Poland (central latitude). Geographic distribution of *Ischnura elegans* in central and northern Europe is shown in grey and occurrence of the spiny‐cheek crayfish *Orconectes limosus* is depicted by red crosses. On the left side, we show the design of the Analysis 1 focusing on different urbanization types (rural and urban populations) from central and high latitude reared at current (20°C) and warming (24°C) temperature. The plot shows the significant latitude × urbanization type interaction for the final larval instar (F‐0) mass with 1 SE. On the right side, we show the design of the Analysis 2 focusing on urban and rural populations from high latitude reared at current (20°C), warming (24°C), and heat wave (28°C) temperature and in a control or a predator cue treatment. The plot shows the significant interaction temperature × predator cue for the growth rate until F‐0 with 1 SE.

Mating pairs of damselflies were caught using insect sweep nets. Adult females of these pairs were collected from two urban and two rural ponds each in southern Sweden (hereafter, high latitude) and in southern Poland (hereafter, central latitude) (Figure 1; Table S1) on 22–23 June 2021 as described in Sniegula et al. (2019). The distances between urban and rural sites ranged from 5 to 19 km at the high latitude and from 26 to 96 km at the central latitude. We quantified the level of urbanization based on the percentage of impervious surface from the high‐resolution layer database (20 m resolution) (EEA, 2020). We created a circular buffer of 1 km around each sampling location and calculated the average value of imperviousness in each buffer using Quantum‐GIS (QGIS Development Team, 2017). We defined two urbanization types: ‘urban’ for ponds with an average percentage of impervious surface area within the buffer above 20% and ‘rural’ when the percentage was below 1.5% (Brans & De Meester, 2018).

Water temperatures in the shallow parts of the collection ponds were estimated using the Lake Model Flake (2009) that closely matches the actual temperature measured in situ (Dinh Van et al., 2014). The modelled temperatures indicated minor differences in water temperature among ponds within‐ and between both latitudes (Figure S1A,B). As the FLake model does not include impervious surface as a parameter and because water temperatures within a pond might vary depending on various parameters such as depth or sun exposure, we also placed temperature loggers (40 cm depth) in five ponds during summer and fall, and in one pond during an entire year (2021 and/or 2022; and Figure S1D). Based on FLake and dataloggers estimates, the average temperature in central‐ and high latitude ponds, and in urban and rural ponds were similar and oscillated around 20°C and were below 24°C during the summer months (except one higher temperature record in one Polish pond, Figure S1C). Based on this and on previous records of freshwater summer water temperatures at these latitudes (Debecker & Stoks, 2019; Dinh Van et al., 2014), we set the following experimental temperatures: 20°C corresponding to the current mean summer water temperature, 24°C corresponding to the predicted increased temperature by 2100 under SSP8.5 scenario (Masson‐Delmotte et al., 2021), and 28°C matching a simulated heat wave.

### 2.3 The crayfish predator

The spiny‐cheek crayfish *Faxonius limosus* is an invasive alien predator and an active colonizer that has locally co‐occurred with the central‐latitude *I. elegans* for at least 50 years but has not been reported in Scandinavia (Artportalen, 2022; Commission Implementing Regulation, 2016; Kouba et al., 2014). The spiny‐cheek crayfish is widely reported in Poland in rural and urban areas (The General Directorate for Environmental Protection, 2018). The spread of the spiny‐cheek crayfish is driven by human activities (aquaculture, trading), and natural spread in Europe (Reynolds & Souty‐Grosset, 2011) and occurs frequently in warm temperatures (up to 25°C) and in polluted waters (Chucholl, 2016). Potential future invasions are more likely to occur in urban areas. Prior the experiment, *F. limosus* were collected from Kryspinów Lake in southern Poland (50°3′0.461″ N, 19°47′20.85″ E) and transported to the Institute of Nature Conservation of the Polish Academy of Sciences, Kraków, Poland (INC PAS). Three crayfishes were kept in an aquarium holding 50 L of dechlorinated tap water along with a control aquarium with 50 L of dechlorinated tap water. Crayfish collection and housing were done with permission from the Regional Directorate for Environmental Protection in Kraków (ref. OP.672.4.2021.GZ).

### 2.4 Experimental procedure

The experimental procedure involved a pre‐treatment part where all larvae experienced the same pre‐winter and winter rearing conditions (hereafter, initial growth period) for all the larvae (Figure S2). After winter (treatment application phase), we started the different thermal treatments and, on the day of entering into the final instar before emergence (F‐0), larvae were exposed to one of the two predator cue treatments for 5 days. The experiment ended at the end of this 5‐day predator cue exposure period.

To obtain eggs, 10 adult females per pond were captured (10 females × two urban ponds × two rural ponds × two latitudes = 80 females). Adult females were individually placed in plastic cups with perforated lids and wet filter paper, and kept at ca. 22°C and natural daylight (photoperiod) for egg laying. In *I. elegans*, egg laying occurs within 3 days. During these 3 days, each female produced one clutch (hereafter, family), hence we used a total of 10 families per pond, and a total of 80 families in the experiment. Then, females were removed and eggs were kept in an incubator at 22°C and a photoperiod of L:D 20:4 h. The photoperiod indicated the longest day length at the high latitude collection site, and was expected to create high development and growth rates in both studied latitudes, especially during post‐winter conditions (Norling, 2021).

For the next step (initial growth period), we placed larvae in group under the same pre‐winter and winter rearing conditions. For each pond, we prepared six containers matching the number of treatments (three temperature and two predator treatments) to be used during the treatment application phase, this resulted in a total of six containers × eight ponds = 48 containers. These plastic containers (size 22 × 16 cm, height 11 cm) were filled with 1500 mL of dechlorinated tap water and all maintained in an incubator at 22°C and L:D 20:4 h. For each pond, once the majority of the eggs of a family hatched, we composed sets of 40 larvae in these six containers in the following way: four larvae from each of the 10 families of a given pond were randomly placed in each of the six containers. This approach ensured that each container started with exactly the same number of individuals from each family. Since the larvae were reared in groups, each container was supplied with a plastic structure to minimise cannibalism among the larvae. Larvae were fed ad libitum with laboratory‐cultured *Artemia nauplii*, twice a day on week days and once a day on weekend days. After 3 weeks of growing, we supplemented the feeding with live *Daphnia* sp. two times a week until autumn conditions. The position of the containers was randomized weekly within each incubator.

On 06 August 2021 (ca. 4 weeks after larvae had hatched), we started simulating autumn temperatures and photoperiods (hereafter, thermo‐photoperiods) and, 3 weeks later, winter conditions. This procedure allowed larvae to experience a winter diapause, as it occurs in nature (Corbet et al., 2006; Norling, 2021). With a weekly interval, we gradually reduced the initial thermo‐photoperiod from 22°C and 20:4 h to 6°C and 0:24 h L:D to simulate autumn and winter conditions in nature. A detailed description of rearing thermo‐photoperiods during the entire experiment are presented in Figure S3. During the simulated winter, larvae were fed three times a week (Monday, Wednesday and Friday) with *Artemia* nauplii.

On 22 November 2022, directly after the simulated winter we started the treatment application phase. First, we transferred all surviving larvae per container to individual 200 mL cups (height = 9 cm, diameter = 4 cm) filled with 100 mL of dechlorinated water and placed each cup into an incubator at 10°C and 4:20 h L:D. With a 2‐day interval, we gradually increased the thermo‐photoperiod to the respective thermal treatment: 20°C (control), 24°C (mild warming) or 28°C (heat wave, high latitude only) with the same 20:4 h L:D photoperiod for all temperature treatments (Figure S3). For logistic reasons we could not set the 28°C temperature treatment group for the central‐latitude populations. We therefore split our design into two analyses: Analysis 1 focusing on high‐ and central latitude populations raised at 20 and 24°C, and Analysis 2 focusing on high‐latitude populations raised at 20, 24 and 28°C.

Throughout the treatment application phase, larvae were fed daily with *Artemia* nauplii. Because larvae came from different latitudes and because rearing temperature also affects larval development rate, larvae reached the F‐0 stage at different dates. This led to larvae being exposed to the post‐winter temperature treatment for different durations. For each larva, we reported the exact time in days at the temperature treatment during the second half of the experiment (‘thermal exposure duration’) and this time was included as a covariate in the statistical models (except for developmental time). In addition, when larvae entered F‐0, we identified the sex of each individual based on absence/presence of emerging signs of an ovipositor under a microscope to test for sex‐specific responses to the treatments (central latitude *N*_females_ = 73, *N*_males_ = 68; high latitude *N*_females_ = 158, *N*_males_ = 182).

When larvae entered the F‐0 stage, we crossed the thermal treatments with a 5‐day‐long predator cue treatment (absence vs. presence). We collected water samples from the crayfish or control aquaria that we warmed up to the target temperature (20, 24 or 28°C). The water level in each cup was reduced to 67 mL and refilled with 33 mL of medium from the crayfish aquarium (with predator cue) or the control aquarium (without predator cue). Cups were refilled every second day to keep the predator cue approximately constant, considering the length of predator cue biodegradation (Van Buskirk et al., 2014). Previous experiments have demonstrated that predator cues affect damselfly life history traits, also in case of short‐time exposure (13 days exposure in Antoł & Sniegula, 2021; 3–9 days exposure in Van Dievel et al., 2016).

### 2.5 Response variables

In total, 481 larvae survived and were phenotyped at the end of the experiment. Details on sample size for each treatment combination and response variable for both analyses are presented in Table S2. When larvae entered F‐0 and before the application of the predator cue treatment, we quantified three traits: development time (DT; number of days between hatching and moult into F‐0), wet mass (mass_F‐0_), and growth rate until F‐0 (GR_F‐0_). Larval wet mass was measured with an electronic balance (Radwag AS.62) and GR_F‐0_ was calculated as ln(mass_F‐0_)/age_F‐0_. After the 5‐day exposure to a predator cue, we measured the wet mass again (mass_final_) and calculated the growth rate over the 5‐day period: GR_final_ = [ln(mass_final_) − ln(mass_F‐0_)]/5, as in McPeek et al. (2001). Values for each phenotypic trait and information on the final sample size in each group are provided in Table S3.

### 2.6 Statistical analyses

All analyses were performed in R (R Core Team, 2013; RStudio Team, 2015). First, we ran a model selection analysis (MuMin r package; Barton, 2023) to select the most appropriate model for each phenotypic variable (DT, mass_F‐0_, GR_F‐0_, and GR_final_). We included in the initial model the following predictors: latitude (only for Analysis 1), sex, temperature, urbanization type, predator (for GR_final_ only), and all the possible interactions; thermal exposure duration was added as a covariate (except for DT because this variable was strongly correlated with exposure duration) and population nested in latitude as a random factor. For each phenotypic variable, model selection analysis was based on the corrected Akaike's information criteria for sample size (AICc) and weights as criteria to determine the best explanatory linear model by keeping only the most relevant predictors and interactions (Table S4).

Given the lack of data for the 28°C treatment for central‐latitude larvae, our overall design was no longer full factorial. We therefore performed two separate full factorial analyses: Analysis 1 focusing on high‐ and central latitude populations raised at 20 and 24°C, and Analysis 2 focusing on high‐latitude populations raised at 20, 24 and 28°C. In Analysis 1, we tested effects of urbanization type (rural vs. urban population), latitude (high‐ and central‐latitude), temperature (20 and 24°C), sex (males and females) and predator cues (for GR_final_ only). We used generalized linear mixed‐effects models (GLMMs). We ran specific models for each variable: DT_F‐0_ with a Poisson distribution, and mass_F‐0_, GR_F‐0_ and GR_Final_ with Gaussian distributions, based on the output of the model selection analysis. The variables were normally distributed. In Analysis 2, we tested for effects of urbanisation type, temperature (20, 24 and 28°C) and predator (for GR_final_ only) in high‐latitude populations. To fit the GLMMs, we used the function glmmTMB (glmmTMB package; Magnusson et al., 2017). P‐values were obtained using the Wald chi‐square test (Wald *χ* ^2^) implemented in the car package (Fox & Weisberg, 2019).

To analyse in detail the multivariate phenotypic responses of the larvae we applied Phenotypic Trajectory Analysis (PTA) following the procedure and R scripts described in Adams and Collyer (2009) and Collyer and Adams (2007). For this analysis, we used the measurements when larvae entered F‐0 (hence, prior to the predator cue treatment). We used PTA to compare trajectories of high‐ and central‐latitude populations in response to urbanization type (evolutionary change expressed as a magnitude of phenotypic difference between rural and urban populations) and to temperature (plastic change expressed as the slope of the thermal reaction norm). We ran PTA both with the sexes pooled, and with males and females separately. The procedure consists of a vector analysis in a multivariate space of phenotypic trait change. First, we conducted a PTA using DT_F‐0_, mass_F‐0_, and GR_F‐0_. To account for the fact that damselflies were exposed to the temperature treatment for different durations, we ran a GLM model for mass_F‐0_ and GR_F‐0_ with ‘thermal exposure duration’ as covariate and extracted the residuals that were subsequently used for PTA analyses. The three variables DT_F‐0_ and the residuals values for mass_F‐0_, and GR_F‐0_ were scaled and centred before running the PTA. Then, we used PCA for visualising and plotting the PTA output. For the PCA, we used the same variables as for the PTA: DT_F‐0_ and the residuals values for mass_F‐0_, and GR_F‐0_ that were scaled and centred. We created vectors connecting centroids of groups of individuals to calculate and compare the difference in length (magnitude) and angle (direction, *θ*) between groups. Significance of the magnitude and direction of the phenotypic response vectors were estimated using a permutation procedure (*N* = 1000 permutations).

## 3 RESULTS

### 3.1 Analysis 1

#### 3.1.1 Univariate response patterns

**FIGURE 2:**
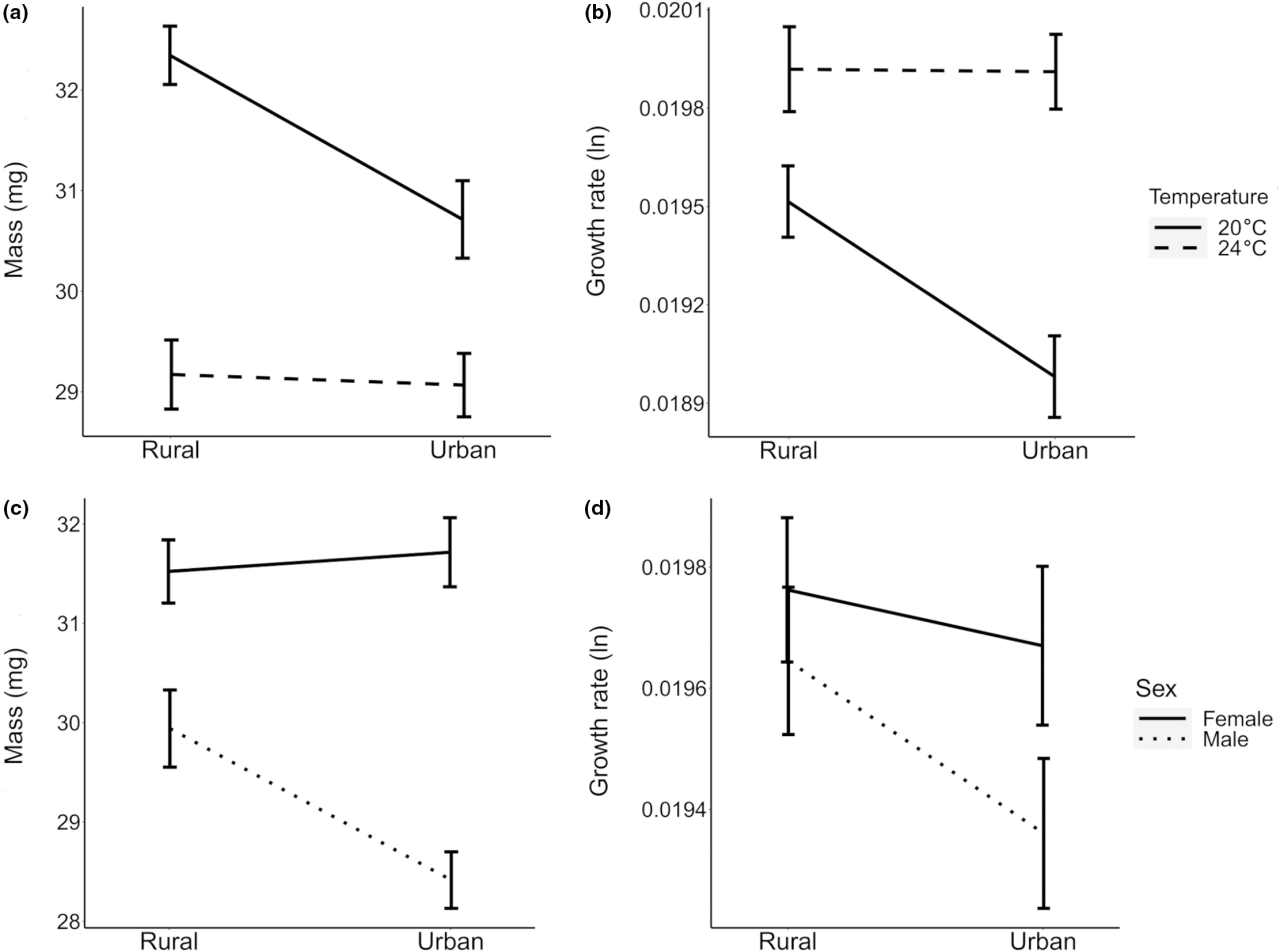
Larval (a) mass_F0_ and (b) growth rate_F0_ (GR_F0_) across urbanization types (urban and rural populations) and current (20°C) and warming (24°C) temperature. Larval (c) mass_F0_ and (d) GR_F0_ across urban and rural populations for females and males in Analysis 1 (*N* = 349). Given are estimated means with 1 SE.

The results testing for the effects of the treatments on DT, mass, and GR are shown in Table 1. Urbanization type had no main effect, but several interactions involving urbanization type were significant. At 20°C, urban larvae had a lower mass_F‐0_ and GR_F‐0_ compared to rural larvae. Mild warming (24°C) reduced mass_F‐0_, especially in rural larvae, and increased GR_F‐0_, especially in urban larvae, eliminating the differences for these traits between urban and rural populations at 24°C (interaction urbanization type × temperature) (Figure 2a,b). While urban and rural females did not differ in mass_F‐0_ and GR_F‐0_, urban males were lighter and showed a lower GR_F‐0_ than rural males (interaction urbanization type × sex; Figure 2c,d). Urbanization type decreased larval mass, but in central‐latitude individuals only (interaction latitude × urbanization type; Figure 1); there was a larger difference in mass_F‐0_ between latitudes in females than in males (interaction latitude × sex; Figure S4A). The interaction latitude × temperature was significant for GR_F‐0_, with higher values of GR_F‐0_ in central‐ compared to high latitude individuals but only at 24°C (Figure S4B). DT was not affected by urbanization type, but independently affected by latitude and temperature, with a shorter development time in central‐latitude larvae and in larvae reared at 24°C (Table S5, Figure S5).

**TABLE 1:** Results of the GLMM of the Analysis 1.

(A)	df	At entrance to F‐0			(B)	df	Predator treatment
		Dev. time_F0_	Mass_F0_	GR_F0_			GR_final_
Variables		*p*	*p*	*p*	Variables		*p*
Thermal exposure duration	1	–	0.001 (10.1)**	0.001 (1150)***	Thermal exposure duration	1	0.001 (10.6)**
Sex	1	–	<0.001 (80.7)***	0.001 (75.2)***	Latitude	1	0.022 (5.25)*
Latitude	1	<0.001 (14.2)***	<0.001 (53.1)***	0.001 (112)***	Predator cue	1	0.004 (8.39)**
Temperature	1	<0.001 (50.0)***	<0.001 (76.7)***	0.001 (62.0)***			
Urbanization type	1	**–**	0.308 (1.03)	0.236 (1.41)			
Latitude × sex	1	**–**	0.036 (4.40)*	0.116 (2.47)			
Latitude × temperature	1	–	0.050 (3.83)	0.017 (5.71)*			
Latitude × urbanization type	1	**–**	0.004 (8.15)**	**–**			
Temperature × sex	1	**–**	0.165 (1.93)	**–**			
Temperature × urbanization type	1	**–**	0.010 (6.70)**	0.047 (3.93)*			
Urbanization type × sex	1	**–**	0.027 (4.87)*	0.032 (4.59)*			

*Note*: Table shows effects of sex (females and males), latitude (central and high), urbanization type (rural and urban), temperature (20 and 24°C) and of their interactions on larval development time, mass and growth rate (a) at entrance to F‐0 (DT_F0_, Mass_F0_ and GR_F0_) and (b) during the 5‐days exposure period to predator cues (GR_final_). For each variable, we limited the analysis to the relevant predictors and interactions determined by the model selection analysis. The duration of the temperature treatment (post‐winter ‘thermal exposure duration’) was included as covariate. Effects of random factors are not shown. Table shows *p*‐values and Wald Chi‐squared statistic (in parentheses) for each variable. Significance is indicated in bold by ***p < 0.001; **p < 0.01; *p < 0.05.

During the 5‐day predator cue treatment, latitude and predator treatment had significant effect on GR_final_, with faster growth rate in central‐latitude larvae and in the absence of predator cues (Table S5, Figure S6). None of the interaction terms were significant for the GR_final_.

#### 3.1.2 Phenotypic trajectory analysis

**FIGURE 3:**
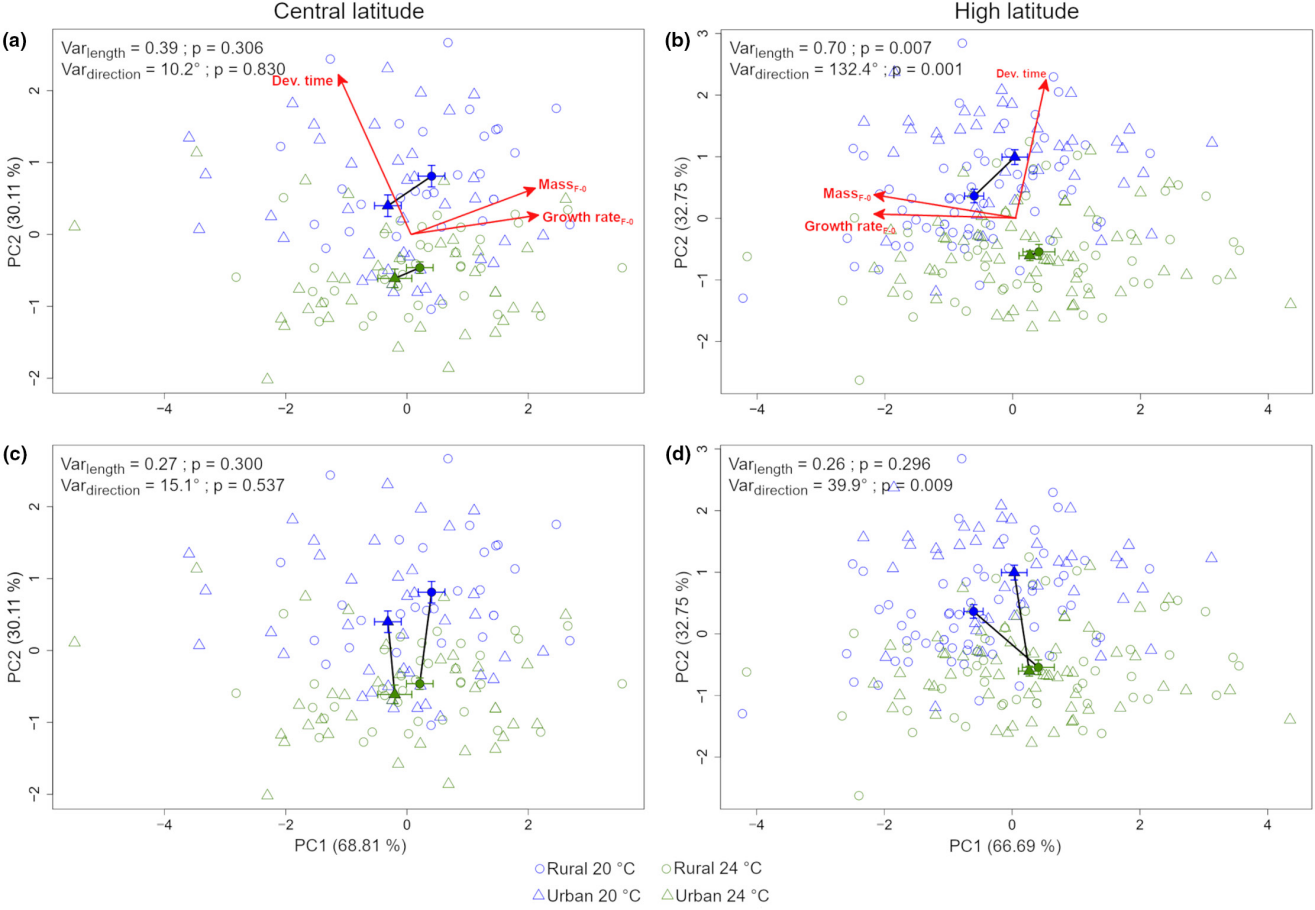
Principal component analysis showing evolutionary changes before the treatment with predator cue in response to urbanization type at current (20°C) and warming (24°C) for (a) central‐ (*N* = 141) and (b) high latitude (*N* = 208) populations and plastic changes in response to temperature in rural and urban populations for (c) central‐ and (d) high latitude populations (sexes pooled). Rural and urban individuals are depicted by open circles and triangles respectively; temperature by colours (blue = 20°C and green = 24°C); filled circles and triangles correspond to the centroid of each group; solid lines connecting filled symbols represent the vector.

For central‐latitude populations, evolutionary changes in response to urbanization type between larvae raised at 20 and 24°C did not differ in length (Var_length_ = 0.39, *p* = 0.306) nor in direction (*θ* = 10.2°, *p* = 0.830). Evolutionary changes driven by urbanization type pointed mostly to a lower mass and growth rate in urban areas (Figure 3a). Plastic changes in response to mild warming temperature between rural and urban populations did not differ in length (Var_length_ = 0.27, *p* = 0.300) nor in direction (*θ* = 15.1°, *p* = 0.537). Urban and rural populations responded in a similar way to mild warming temperature with a shorter development time when temperature increased (Figure 3c).

Next, we ran similar analyses for males and females separately. For males, we found the same patterns for evolutionary and plastic changes as with the sexes pooled (Figure S7A,C). For females, we found different evolutionary trajectories (differences in direction only) between females raised at 20 and 24°C (Figure S8A); at 20°C evolutionary changes in response to urbanization type pointed to a decrease in mass and GR_F‐0_ and the pattern was reversed at 24°C. Thermal plasticity did not differ between rural and urban females (Figure S8C).

For high‐latitude populations, when sexes were pooled, evolutionary changes in response to urbanization type differed both in length (Var_length_ = 0.70, *p* = 0.007) and direction (*θ* = 132.4°, *p* = 0.001) between the two temperature treatments (Figure 3b). Urban larvae had a longer DT, and lower mass and GR than rural larvae at 20°C, whereas there was little difference between urban and rural damselflies at 24°C. Plastic changes in response to an increase in temperature showed no difference in magnitude between rural and urban populations (Var_length_ = 0.26, *p* = 0.296) but significant differences in direction (*θ* = 39.9°, *p* = 0.009) with a decrease in mass and GR at 24°C only being present in rural populations (Figure 3d).

When looking at each sex separately, for males, we found the exact same patterns for evolutionary (Figure S7B) and plastic (Figure S7D) changes as with the sexes pooled. For females, evolutionary changes differed only in direction between the temperature treatments; at 20°C evolutionary changes pointed to an increase in development time in urban area and at 24°C to an increase in mass and GR_F‐0_ (Figure S8B). Plastic changes to mild warming temperature for females followed the same pattern as with the sexes pooled (Figure S8D).

### 3.2 Analysis 2

#### 3.2.1 Univariate response patterns

In the set of high‐latitude urban and rural larvae tested at the three temperatures, the score life‐history traits (mass_F0_, DT_F0_, and GR_F0_) were not affected by urbanization type, but were affected by temperature and sex (Table 2; Table S4). The interaction temperature × sex was significant for both mass_F‐0_ and GR_F‐0_, with higher values in mass_F‐0_ and GR_F‐0_ in females, especially at 24°C (Figure S9). GR_final_ was not affected by urbanization type. Exposure to the predator cue during the 5‐day‐long treatment reduced GR_final_ at 20 and 24°C, but instead increased GR_final_ at 28°C (predator × temperature; Figure 1).

**TABLE 2:** Results of the GLMM of the Analysis 2.

(A)	df	At entrance to F‐0			(B)	df	Predator treatment
		Dev. time_F0_	Mass_F0_	GR_F0_			GR_final_
Variables		*p*	*p*	*p*	Variables		*p*
Thermal exposure duration	1	–	<0.001 (34.3)***	**<0.001 (1168**)***	Thermal exposure duration	1	<0.001 (12.5)***
Sex	1	0.029 (4.78)*	<0.001 (91.1)***	<0.001 (84.8)***	Temperature	2	0.122 (4.21)
Temperature	2	<0.001 (43.0)***	<0.001 (324)***	<0.001 (316)***	Predator cue	1	0.344 (0.89)
Urbanization type	1	0.063 (3.43)	**–**	**–**	Temperature × predator cue	2	0.001 (13.0)**
Temperature × sex	2	**–**	0.020 (7.83)*	0.026 (7.29)*			
Temperature × urbanization type	2	0.31 (2.36)	**–**	**–**			

*Note*: Table shows effects of sex (females and males), urbanization type (rural and urban), temperature (20, 24 and 28°C) and of their interactions on larval development time, mass and growth rate (a) at entrance to F‐0 (Dev. time_F0_, Mass_F0_ and GR_F0_) and (b) during the 5‐days exposure period to predator cues (GR_final_). For each variable, we limited the analysis to the relevant predictors and interactions determined by the model selection analysis. The duration of the temperature treatment (post‐winter ‘thermal exposure duration’) was included as covariate. Effects of random factors are not shown. Table shows *p*‐values and Wald Chi‐squared statistic (in parentheses) for each variable. Significance is indicated in bold by ***p < 0.001; **p < 0.01; *p < 0.05.

#### 3.2.2 Phenotypic trajectory analysis

**FIGURE 4:**
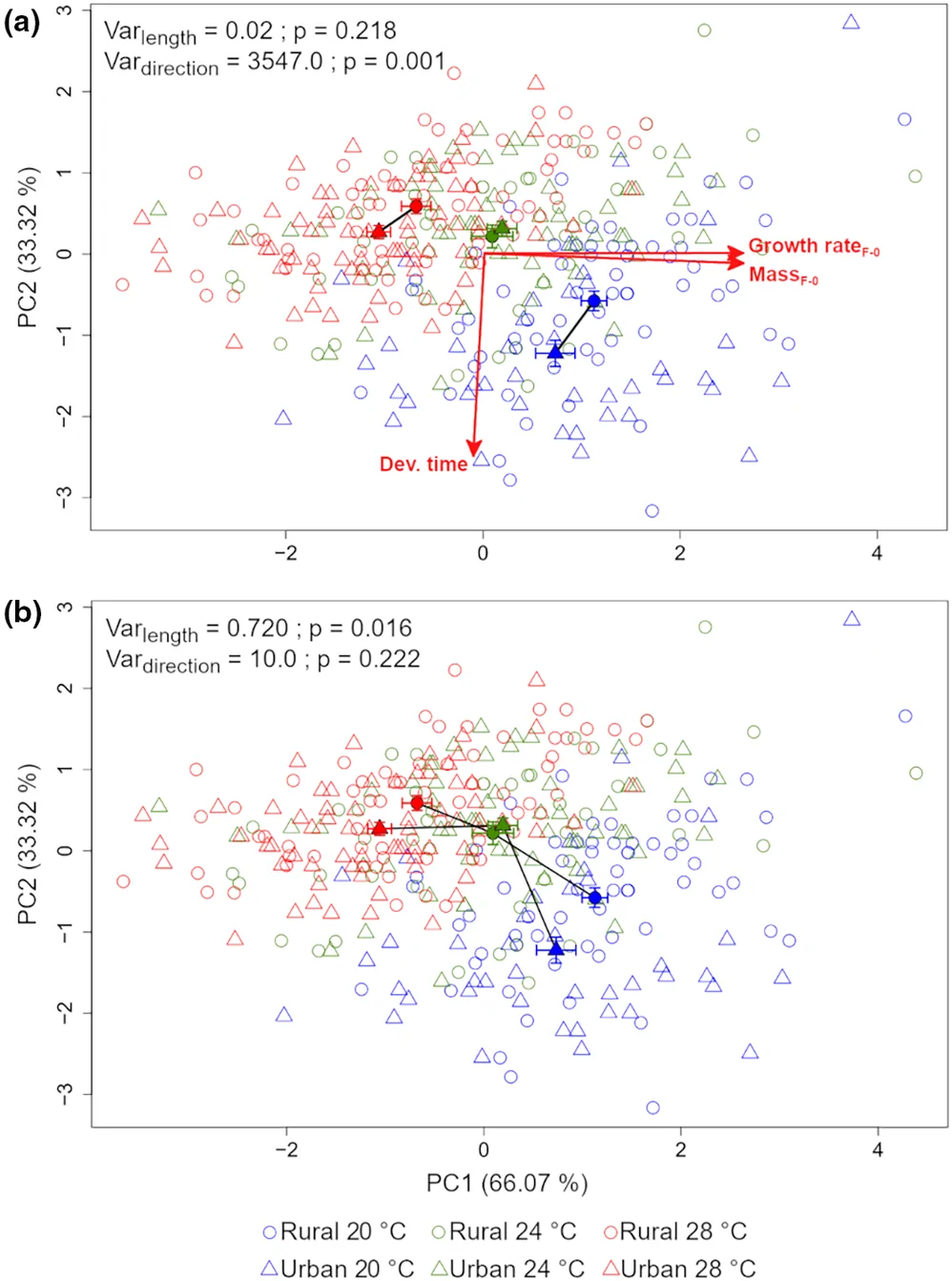
Phenotypic plasticity trajectory in response to (a) urbanization type at current (20°C), warming temperature (24°C) and heat wave (28°C) and to (b) temperature for rural and urban populations (*N* = 340). Rural and urban individuals are depicted by open circles and triangles respectively; temperature by colours (blue = 20°C, green = 24°C and red = 28°C); filled circles and triangles correspond to the centroid of each group; solid lines connecting filled symbols represent the vector.

Phenotypic trajectory analysis showed that high‐latitude populations exhibited different evolutionary changes in response to urbanization type across the three temperature treatments (sexes pooled). We did not find significant variation in magnitude between the three vectors (Var_length_ = 0.02, *p* = 0.218) but we found significant differences in their direction (Var_direction_ = 3547.0, *p* = 0.001) (Figure 4a). At 20 and 28°C, urbanization type is mostly accompanied by longer development and lower mass_F‐0_ and GR_F‐0_, whereas at 24°C differences between rural and urban individuals were rather small. For plastic changes, PTA revealed significant differences in length (Var_length_ = 0.720, *p* = 0.016) but not in direction (Var_direction_ = 10.0, *p* = 0.222) between the two vectors indicating a greater thermal plasticity in urban than in rural populations (Figure 4b).

When looking at each sex separately, evolutionary changes in response to urbanization type were similar for males and females and followed the same patterns as with the sexes pooled (Figure S10A,B). For plastic changes, no difference in both length and direction was found in males (Figure S10C), whereas differences in length were found in females (Figure S10D); with a greater thermal plastic response in females from urban than rural populations.

## 4 DISCUSSION

We studied life history adaptation in an ectotherm in response to urbanisation at different latitudes. Our results pointed to effects of urbanization type in combination with temperature on mass and GR. Notably, the direction and magnitude of the evolutionary and plastic responses to temperature in life history traits (i.e. body mass and GR) were not uniform across two the latitudes. The response to a predator was rather independent of the two latitudes studied and of the urbanization type. Our results shed light on the effect of interacting anthropogenic factors in shaping life‐history of an ectotherm and how these patterns may depend on the latitude of origin of the population.

### 4.1 Effects on individual traits

In Analysis 1, we found negative effects of urbanization on larval growth rate and mass, but these effects differed between latitudes and depended on temperature. This partially confirmed that urbanization can decrease mass in insects (Merckx, Souffreau, et al., 2018), and that mass decrease is temperature‐dependent (Diamond et al., 2014). Mild warming was sufficient to remove the differences between rural and urban larval mass and growth rate. Intriguingly, these two traits showed higher thermal plasticity at different urbanization types. While mass showed steeper thermal reaction norm in rural populations, GR was more plastic in urban populations. We might hypothesise that the effect of temperature was stronger than the effect of urbanization type and that growth rate reached some physiological limits at 24°C (growth rate did not increase at 28°C in high latitude populations), however, the pattern was less clear for the mass.

Notably, the effects of urbanization type varied between central and high latitudes. Central‐ and high‐latitudes populations differ in their life‐history strategies. For instance, at central latitudes, the species produces, on average, one generation per year more than at high latitudes (Corbet et al., 2006; Norling, 2021). The increased voltinism in central latitude populations is a consequence of faster larval development and growth which results in lower body mass in central‐ than in high‐latitude populations (Debecker & Stoks, 2019). We may hypothesis that such differences may play role in triggering a differential response to urbanization between the two latitudes. Higher development and growth rates indeed often come at a cost of having a smaller mass at emergence (Raczyński et al., 2022; Sniegula et al., 2018). In the current study, latitudinal differences in mass seemed to be amplified by urbanization type. Indeed, with climate change, there might be a further increase in time constraints in urban areas for central‐latitude individuals that might arose from the heat‐island effects (Chick et al., 2019). However, based on our field measurements, contemporary water temperatures in urban and rural ponds differed slightly (Figure S1), and it is difficult to predict whether climate change will increase this difference in temperature. We may hypothesize that these latitude‐specific effects of urbanization may be magnified towards lower latitudes where populations are even more time‐constrained (Corbet et al., 2006; Diamond et al., 2023; Norling, 2021).

Independently of urbanization type and latitude, the predator cue negatively affected larval GR. In insects, exposure to a predator alters metabolism (Cinel et al., 2020) and foraging activity (Kohler & McPeek, 1989), with downstream effects on larval development and growth rate. Our results indicate that both central‐ and high‐latitude populations reacted in a similar way to the predator cue in the traits we measured. These results contrast with a recent study showing a differential response in development time in *I. elegans* eggs when exposed to cues from non‐native invasive predators (spiny‐cheek and signal crayfish *Pacifastacus leniusculus*) (Antoł & Sniegula, 2021). However, an exposure to a phylogenetically related predator species, i.e. noble crayfish (*Astacus astacus*) present at both latitudes (Kouba et al., 2014), might enable predator cue recognition and trigger similar responses between the two latitudes (Anton et al., 2020).

Results from Analysis 2 did not provide evidence that urban and rural populations coped differently with a simulated heat wave, as previously demonstrated in damselflies (Tüzün & Stoks, 2021) and other ectotherms (Brans et al., 2017; Campbell‐Staton et al., 2020). This may stem from the minor mean temperature differences between urban and rural ponds at our study sites or different experimental approaches. Yet, we found a significant effect of temperature in combination with predator cues. Damselflies grew faster in the absence of a predator cue, but only at the current and warming temperature. Interestingly, exposure to the predator cues increased larval growth under a heat wave treatment. A combination of stressors may change the strategy how prey escape predation (Warkentin, 2011). Here, an accelerated larval growth in the presence of a predator cue under the heat wave may be part of an escape strategy to reduce the time of exposure to predators, as previously shown in odonate species (Antoł & Sniegula, 2021; Stoks et al., 2012) and other taxa (Chivers et al., 2001).

In both analyses, we found multiple sex‐specific effects on mass and GR with greater phenotypic differences between urbanization types and temperatures in males. These results supported previous studies in which sex‐specific effects were found in ectotherms coping with various stressors, i.e. urbanization (Kaiser et al., 2016) and heat stress (Sniegula et al., 2017). Sex‐specific effects are generally more pronounced in species with strong sexual dimorphism which is the case in damselflies, with females being usually larger and heavier than males (Corbet, 1999). Sex‐specific effects are also common in protandrous species with a strong selection acting on males to emerge before females in order to maximise their mating opportunities (Badyaev, 2002). Hence, different selective pressures resulting in different life‐history strategies and associated resource allocation patterns between males and females may lead to different types of trade‐offs. For instance, female butterflies maintained a relative high body mass under different thermal conditions compared to males because mass was more important for females in reproduction than for males (Fischer & Fiedler, 2000). This is likely to be the case in odonates (Sokolovska et al., 2000) and matched our observations of mass of females being less affected by urbanization type and temperature than males (at least until 24°C; Figure S3A).

### 4.2 Multivariate approach

Our results indicate multivariate evolutionary change associated to urbanization type at the high latitude since larvae from urban and rural sites differed in life history traits and in their thermal plasticity. In contrast, central‐latitude damselflies exhibited similar direction and magnitude of evolutionary trajectories in response to urbanization type in the two temperature treatments (Analysis 1). Similar results were found in high‐latitude individuals in response to urbanization type and additional simulation of heat wave (Analysis 2). The ‘pace‐of‐life’ syndrome predicts a shift towards a fast‐living strategy when urbanization type increases or towards lower latitudes, which was supported empirically in birds and invertebrates (Brans & De Meester, 2018; Charmantier et al., 2017; Debecker et al., 2016). We observed that only high latitude damselflies expressed a consistent decrease in mass and growth rate associated with urbanization at 20°C, which was further accompanied with an increase of development time. Hence, we found no support for a faster pace‐of‐life in the studied populations. One explanation might be the minor difference in water temperatures recorded in ponds from which rural and urban *I. elegans* were collected. However, in the damselfly *Coenagrion puella* a lower growth rate in urban compared to rural populations was also shown, despite water temperatures being up to 3.5°C higher in urban than in rural ponds (Tüzün & Stoks, 2021). In that study, the population‐specific growth pattern was explained by relatively lower temperature and hence shorter growing seasons in rural populations, i.e. compensation to time constraints by increased growth rate. Notably, we demonstrated sex‐specific evolutionary trajectories in response to urbanization at both latitudes. Therefore, the population origin (latitude) and the sex, which entail different life‐history strategies or trade‐offs, were more likely to trigger different trajectories than a shared response to urbanization.

Increased levels of environmental disturbance, including stress linked to suboptimal temperatures, in cities is expected to favour phenotypic plasticity (Alberti, Correa, et al., 2017; Alberti, Marzluff, & Hunt, 2017; Kotze et al., 2022). In contrast, rural and urban populations from the central latitude responded with similar magnitude and direction to mild warming by decreasing developmental time, whereas high‐latitude populations showed similar plasticity in this trait to mild warming in terms of magnitude, but not in terms of direction. However, in response to the heat wave, we did find a greater magnitude of the plastic response in urban populations for GR_F‐0_ and mass, matching the expectation of an increased phenotypic plasticity in urban populations. Different effects of urbanization on plasticity between the two latitudes may be partially caused by different life history strategies: lower latitude organisms tend to have shorter generation times (Corbet et al., 2006). Based on these results, we may infer that not only ‘urban heat island’ effects impose differential selective pressures on organisms across urbanisation gradients (Diamond et al., 2023; Shochat et al., 2006), but also geographic origin and temperature exposition in rural and urban populations have the potential to adjust the damselfly phenotype.

## 5 CONCLUSION

Despite the accumulating evidence that urbanization shapes phenotypes, and the expectation these evolutionary and plastic responses may differ among latitudes, the latter has rarely been tested. We showed that the damselfly responses to urbanization differed between the two latitudes. Notably, the latitude‐specific response to urbanization was also temperature‐dependent, which could be explained by differences in life‐history strategies across latitudes. Both urban and rural populations had the potential to produce a plastic response to warming, yet the magnitude and direction of the plastic changes differed between latitudes. Moreover, we added to the knowledge that the response to urbanization can differ between sexes. In contrast, urban and rural damselflies responded similarly to the presence of a predator cue, the later interacting with different temperatures. Our results highlight the context‐dependency of evolutionary and plastic responses to urbanisation, and caution for generalizing of how populations respond to cities based on populations at a single latitude.

## CONFLICT OF INTEREST STATEMENT

The authors declare no conflicts of interest.

## Supporting information


File S1

Figure S1

Figure S7

Figure S8

Figure S10

Table S3

Table S4

## Data Availability

The dataset containing the phenotypic data is included in Table S3 and is available on Figshare https://doi.org/10.6084/m9.figshare.23708748.v2 (Palomar et al., 2023).
