# Supplementary material for: Latitude‐specific urbanization effects on life history traits in the damselfly Ischnura elegans
Source: Evol Appl. 2023 Aug 1;16(8):1503–15. doi: 10.1111/eva.13583 (PMC10445092; doi:10.1111/eva.13583)
Supplement: Supplementary file 1 — File S1 [file EVA-16-1503-s004.docx]

**Electronic Supplementary Material**

**Latitude-specific urbanization effects on life history traits in the damselfly *Ischnura elegans­***

Separate files

**Fig. S1**. Monthly temperatures for each Swedish (SW; high latitude) and Polish (PL; central latitude) ponds across urban and rural landscapes depicted by the type of lines (solid = rural; broken = urban). The figure shows temperatures for (A) central- and (B) high latitude ponds estimated using Flake (Lake Model Flake 2009) over the period 1998 – 2009. FLake model does not consider impervious surface as a parameter. Dataloggers were installed (ca. 40 cm depth) in each pond for one year or only several months spanning the summer period in (C) central- and (D) high latitude ponds. For the urban pond located in Krakow (panel C), we placed two dataloggers, one exposed to the sun “Krakow (PL) - sun” and one in shady area “Krakow (PL) - shade” to catch the variability of temperatures within a single pond.

**Fig. S7**. Principal component analysis (PCA) conducted on males only and showing evolutionary changes in response to urbanization at current (20 °C) and mild warming (24 °C) temperature for (A) central- and (B) high-latitude populations and plastic changes in response to different temperatures in rural and urban populations for (C) central- and (D) high-latitude populations. The PCAs were run on response variables measured at the larval entrance into the final instar, and before the treatment with predator cue. Rural and urban individuals are depicted by open circles and triangles, respectively; temperature by colours, blue = 20 °C and green = 24 °C; filled circles and triangles correspond to the centroid of each group; solid lines connecting filled symbols represent the vector.

**Fig S8**. Principal component analysis (PCA) conducted on females only and showing evolutionary changes in response to urbanization at current (20 °C) and mild warming (24 °C) temperature for (A) central- and (B) high-latitude populations and plastic changes in response to different temperatures in rural and urban populations for (C) central- and (D) high-latitude populations. The PCAs were run on response variables measured at the larval entrance into the final instar, and before the treatment with predator cue. Rural and urban individuals are depicted by open circles and triangles, respectively; temperature by colours, blue = 20 °C and green = 24 °C; filled circles and triangles correspond to the centroid of each group; solid lines connecting filled symbols represent the vector.

**Fig S10**. Principal component analysis (PCA) showing evolutionary changes in response to urbanization at current (20 °C), mild warming temperature (24 °C) and heat wave (28 °C) for (A) males and (B) females and plastic changes in response to temperature in rural and urban populations for (C) males and (D) females from high-latitude populations. The PCAs were run on response variables measured at the larval entrance into the final instar, and before the treatment with predator cue. Rural and urban individuals are depicted by open circles and triangles respectively; temperature by colours (blue = 20 °C, green = 24 °C and red = 28 °C); filled circles and triangles correspond to the centroid of each group; solid lines connecting filled symbols represent the vector.

**Fig. S2.** Summary of the experimental procedure. A) We collected 10 females (= 10 families) per urban and rural ponds (different urbanization types) at central and high latitudes. Females were kept in individual plastic jars for egg laying, each clutch represents a family. Then, for each pond, we prepared six plastic containers that would later correspond to the different treatments (20ºC control, 20ºC predator cue, 24ºC control, 24ºC predator cue, 28ºC control, and 28ºC predator cue) (6 containers x 8 ponds = 48 containers in total). For each pond, once the majority of the eggs of a family hatched, we divided the larvae in these six containers in the following way: four larvae from each family were randomly placed in each of the six containers, totalling 40 larvae per container (4 larvae x 10 families). Larvae were kept in these containers for pre-winter and winter conditions. After winter, larvae were individualized in plastic cups and the experimental (temperature and predator cues) treatments started. B) Details on the application phase. Once larvae were split into individual cups, we started the temperature treatment. When larvae entered the final instar prior emergence (F-0), we crossed the thermal treatments with a five-day-long predator cue treatment (absence vs presence). The water level in each cup was reduced to 67 mL and refilled with 33 mL of water sample medium from the crayfish aquarium (with predator cue) or the control aquarium (without predator cue). On the right side of the figure, we indicated the final sample size after considering that some larvae died during the treatment application phase.

**
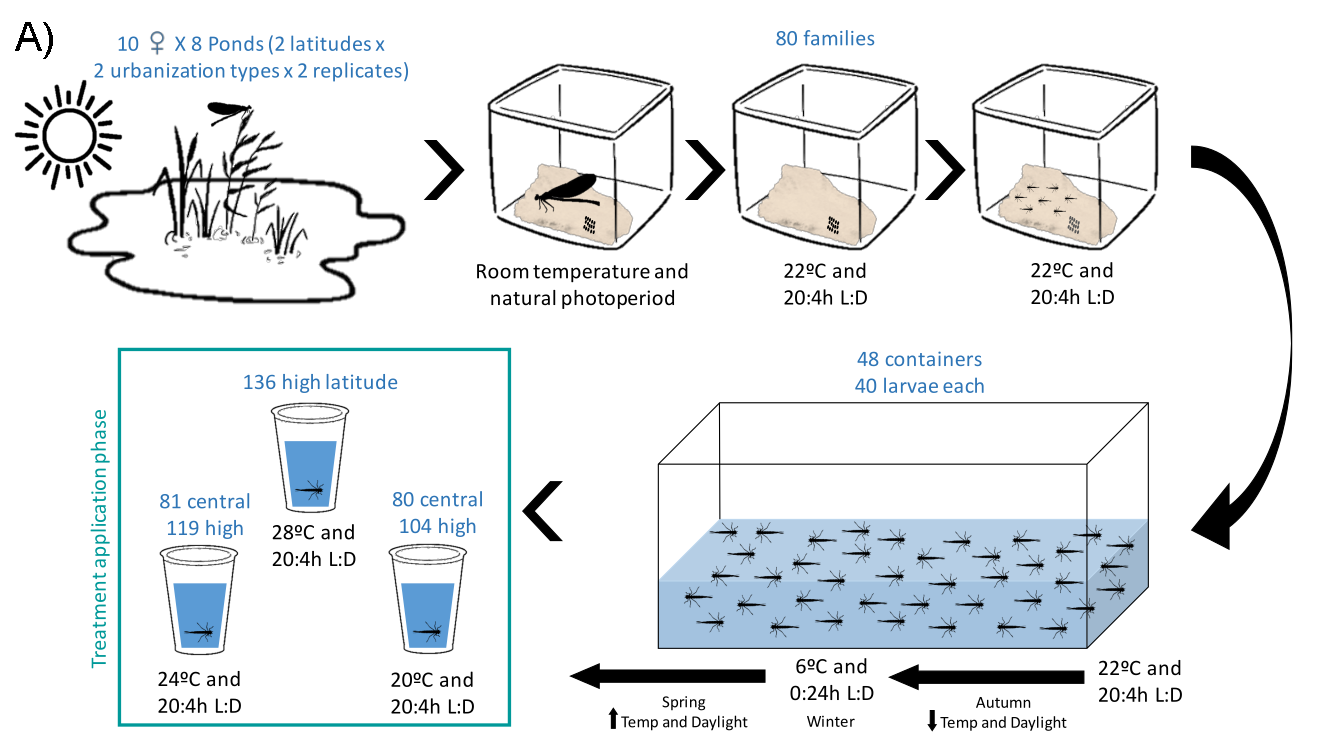
**

**
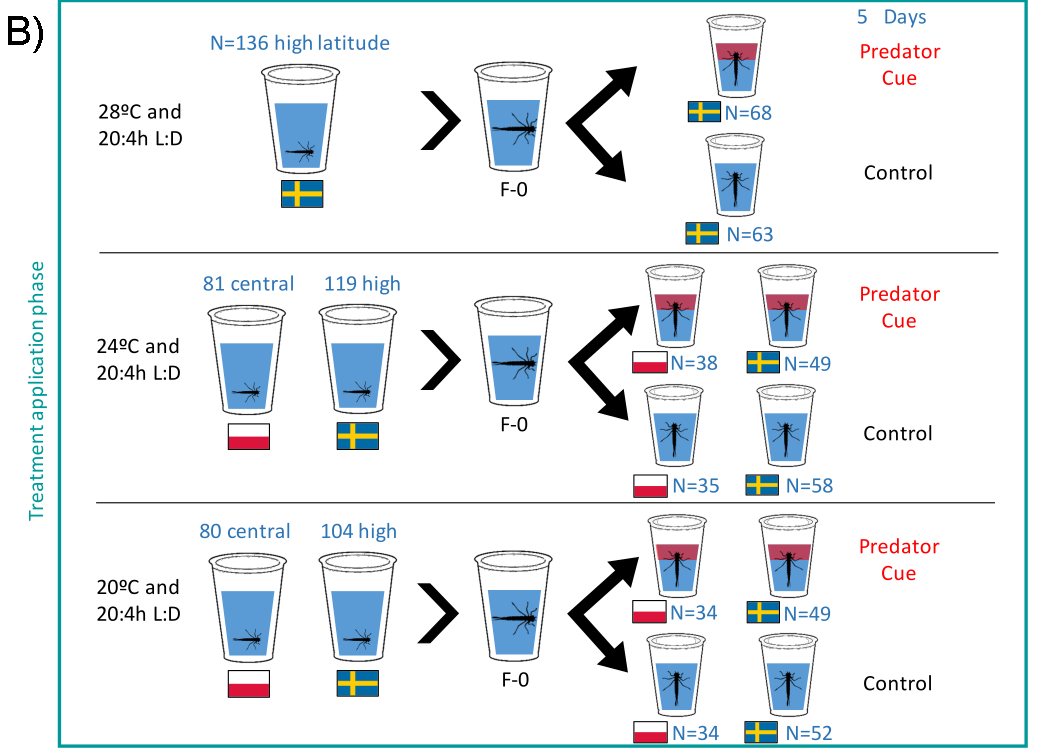
**

**Fig. S3**. Details on (A) rearing temperatures and (B) photoperiod (green line) used over the course of the experiment. Panel A indicates the wintering period (grey area; 6 ºC constant). The end of each curve to the right indicates the end of the experiment, i.e. when last F-0 larva ended the five-day-long predator cue treatment.


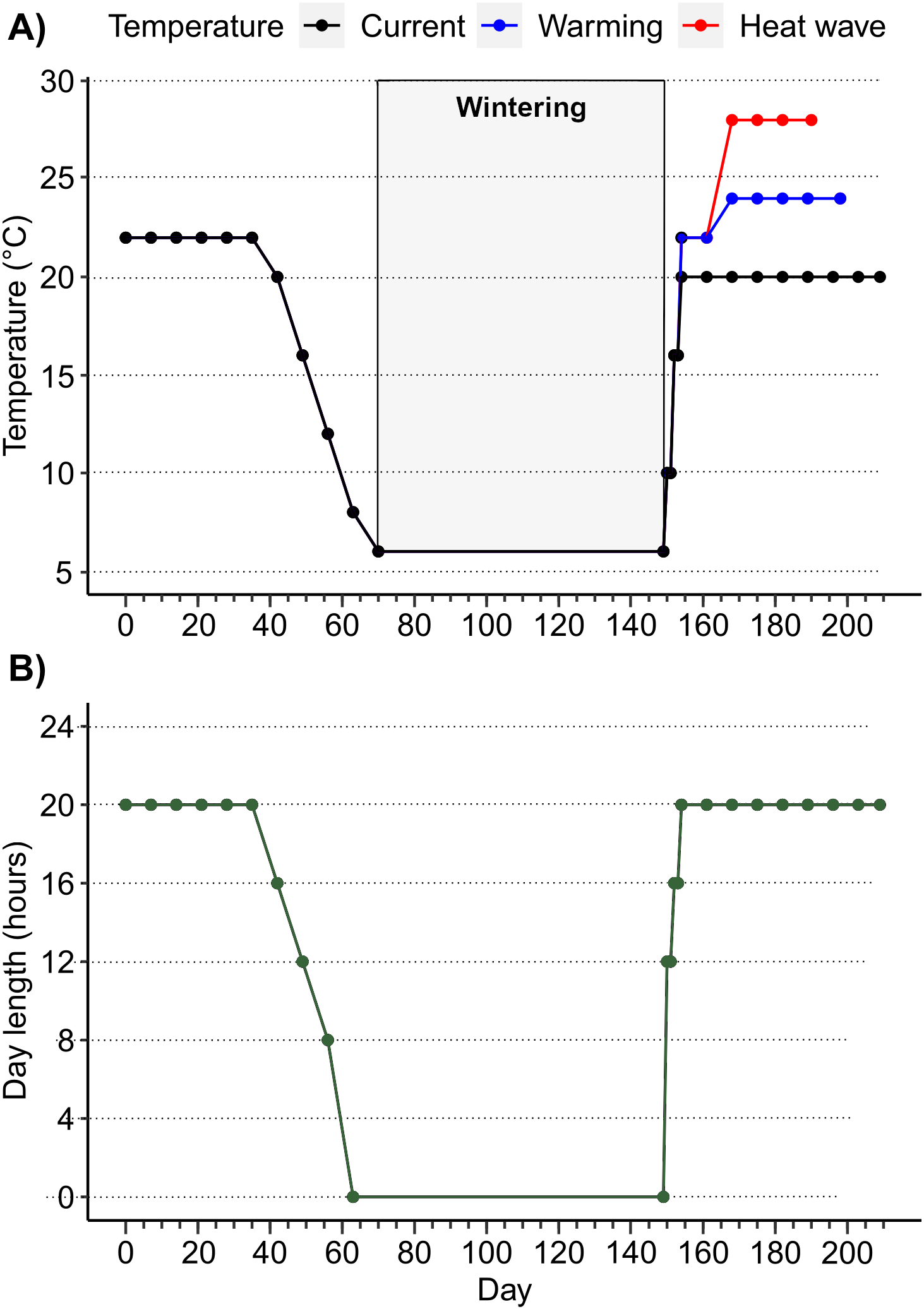


**Fig. S4**. Larval (A) mass _F0_ of females and males and (B) growth rate _F0_ in 20 °C and 24 °C, for central- and high latitude populations in Analysis 1.


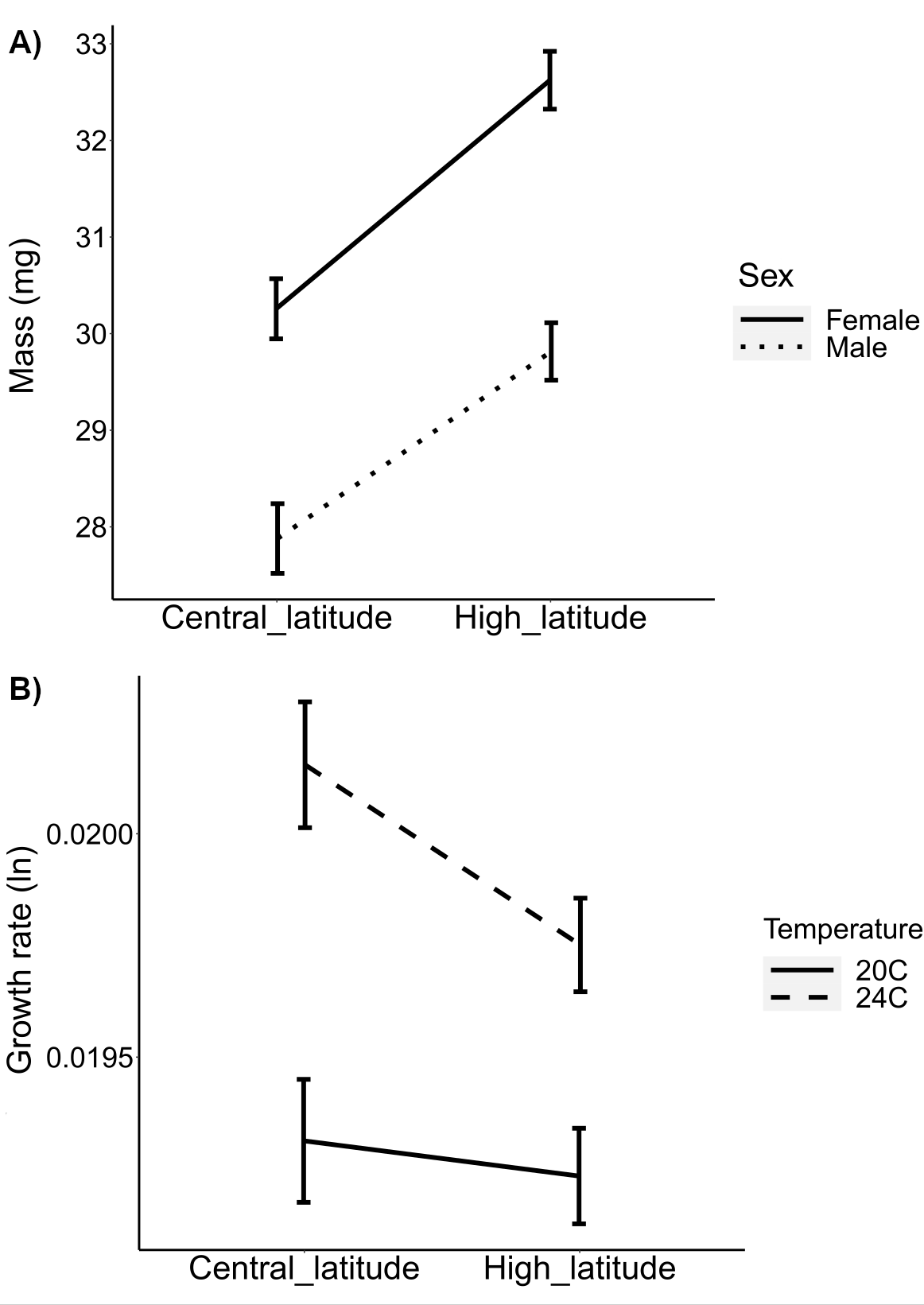


**Fig. S5.** Larval development time (in days) for central- and high latitude populations in different temperature treatments. Black horizontal lines represent the median and the box the 25th and 75th percentiles. Vertical bars are for standard errors and outliers are represented by dots.

**
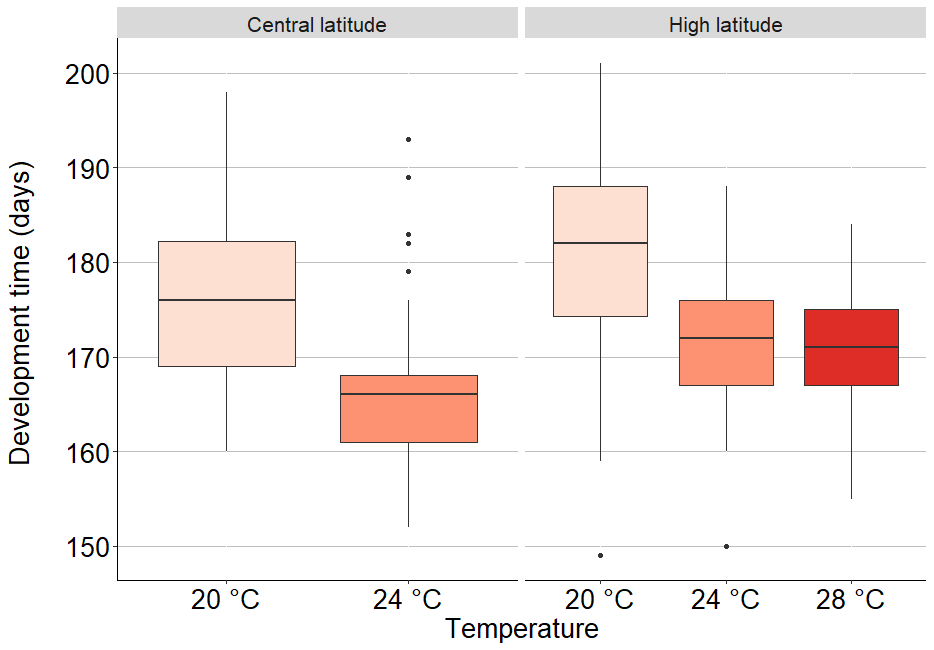
**

**Fig. S6.** Growth rate _final_ (ln) for A) central- and high-latitude populations and B) each predator treatment (absence vs. presence of a predator cue) for Analysis 1. Black horizontal lines represent the median and the box the 25th and 75th percentiles. Vertical bars are for standard errors and outliers are represented by dots.

**
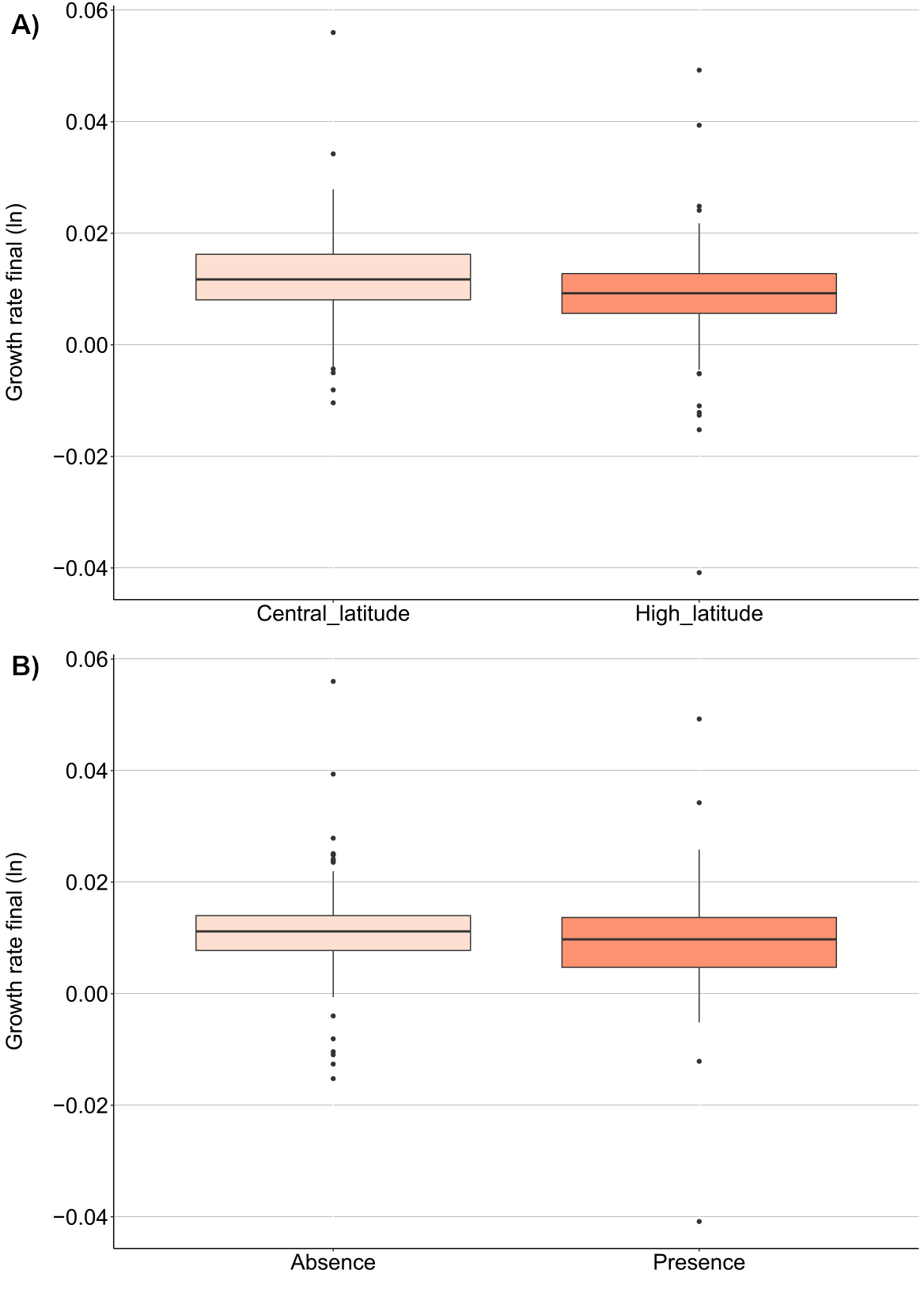
**

**Fig. S9**. Larval (A) mass _F0_ and (B) growth rate _F0_ (GR _F-0_) at current (20 °C), mild warming (24 °C) and heat wave (28 °C) temperature for females and males in Analysis 2.


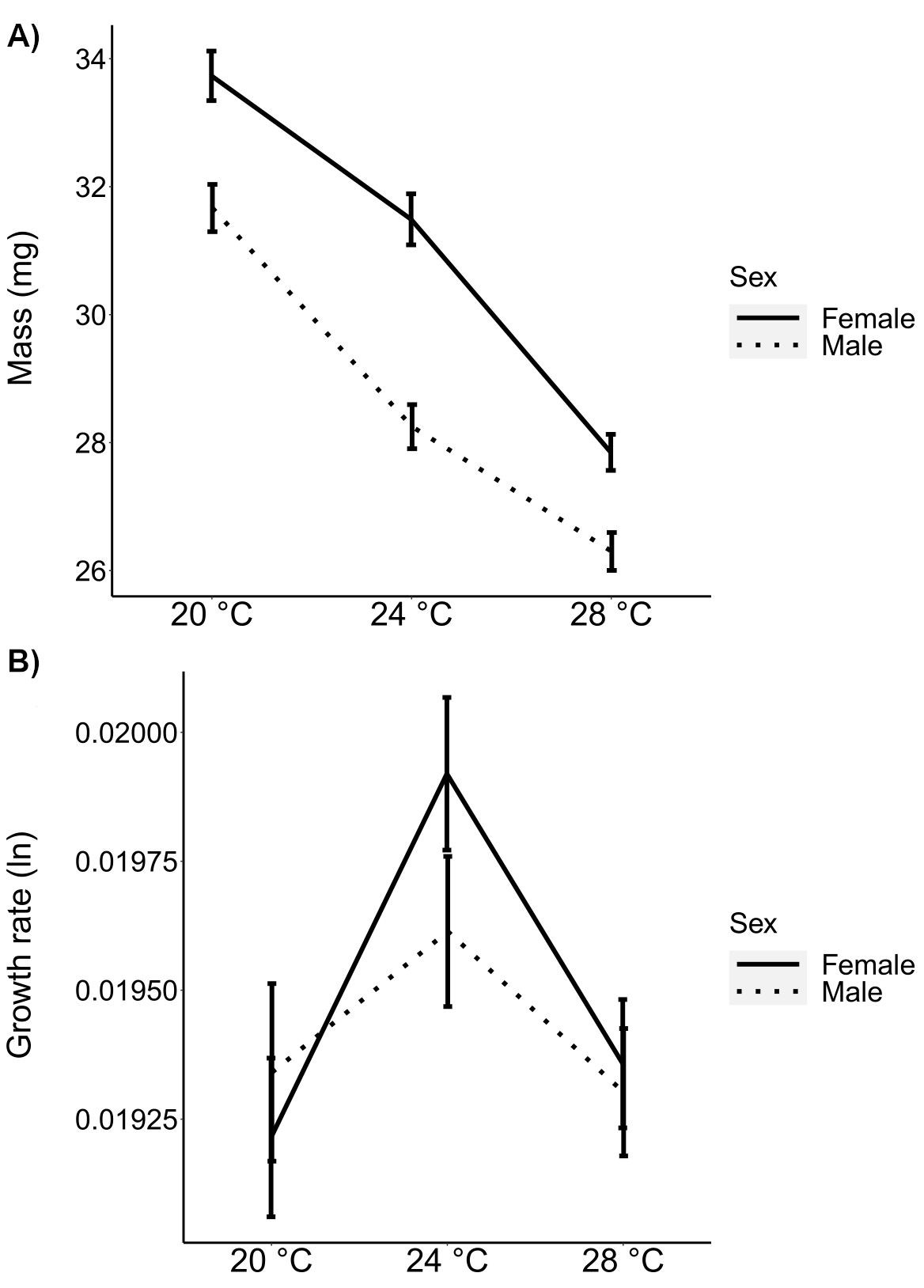


**Table S1**. Description of the sampled ponds.

|  |  |  |  | **Urbanization** | | **GPS coordinates** | |
| --- | --- | --- | --- | --- | --- | --- | --- |
| **Locality** | **Country** | **Latitude** | **Elevation (m)** | **Type** | **% impervious surface** | **Latitude** | **Longitude** |
| Torups | Sweden | High | 32.0 | Rural | 1.35 | 55.564293 | 13.205614 |
| Vallkärra | Sweden | High | 24.0 | Rural | 0.19 | 55.738166 | 13.153274 |
| Kronoborg | Sweden | High | 4.0 | Urban | 38.15 | 55.591598 | 12.993968 |
| Lund | Sweden | High | 24.0 | Urban | 25.07 | 55.705091 | 13.203357 |
| Zagorze | Poland | Central | 275.0 | Rural | 0.37 | 50.083352 | 19.39736 |
| Niepolomice | Poland | Central | 192.0 | Rural | 0.00 | 50.10875 | 20.348707 |
| Krakow | Poland | Central | 201.0 | Urban | 24.19 | 50.064616 | 19.986129 |
| Katowice | Poland | Central | 279.0 | Urban | 32.76 | 50.280691 | 19.021941 |

**Table S2**. Sample size (*N*) per explanatory variable in Analysis 1 and Analysis 2 at the start of post-winter treatments.

|  |  | **By variable** | | | | | | | | | | | | |
| --- | --- | --- | --- | --- | --- | --- | --- | --- | --- | --- | --- | --- | --- | --- |
| **Analysis 1** | **Total** | **Latitude** | | **Urbanization** | | **Sex** | | **Temperature** | | | | | **Predator cue** | |
|  |  | Central | High | Rural | Urban | Male | Female | 20 ºC | | | 24 ºC | | Presence | Absence |
| *N* | 349 | 141 | 208 | 174 | 175 | 179 | 170 | 169 | | | 180 | | 179 | 170 |
|  |  |  |  |  |  |  |  |  | |  | | |  |  |
| **Analysis 2** | **Total** | **Latitude** | | **Urbanization** | | **Sex** | | **Temperature** | | | | | **Predator cue** | |
|  |  | High | | Rural | Urban | Male | Female | 20 ºC | 24 ºC | | | 28 ºC | Presence | Absence |
| *N* | 340 | 340 | | 173 | 167 | 182 | 158 | 101 | 107 | | | 132 | 174 | 166 |

**Table S5**. Least square mean values and standard errors for each response variable in Analysis 1 and Analysis 2.

| Analysis 1 |  | Entrance into the final instar F-0 | | | Predator cue treatment |
| --- | --- | --- | --- | --- | --- |
|  |  | Dev. time (days) | Mass (mg) | Growth rate (ln) | Growth rate (ln) |
| **Sex** | **Male** | 173 ± 0.73 | 29.1 ± 0.23 | 19.5e-3 ± 8.7e-5 | 10.6e-3 ± 6.1e-4 |
|  | **Female** | 175 ± 0.73 | 31.6 ± 0.24 | 19.7e-3 ± 8.9e-5 | 9.8e-3 ± 6.2e-4 |
| **Latitude** | **Central** | 171 ± 0.80 | 29.1 ± 0.27 | 19.7e-3 ± 9.8e-5 | 12.2e-3 ± 6.8e-4 |
|  | **High** | 176 ± 0.66 | 31.1 ± 0.22 | 19.5e-3 ± 8.0e-5 | 8.8e-3 ± 5.6e-4 |
| **Temperature** | **20 ˚C** | 179 ± 0.65 | 31.6 ± 0.24 | 19.3e-3 ± 8.6e-5 | 9.7e-3 ± 6.3e-4 |
|  | **24 ˚C** | 169 ± 0.63 | 29.1 ± 0.23 | 19.9e-3 ± 8.4e-5 | 10.8e-3 ± 6.1e-4 |
| **Urbanization type** | **Rural** | 174 ± 0.74 | 30.8 ± 0.25 | 19.7e-3 ± 8.8e-5 | 10.6e-3 ± 6.2e-4 |
|  | **Urban** | 174 ± 0.74 | 29.8 ± 0.25 | 19.5e-3 ± 8.8e-5 | 9.8e-3 ± 6.2e-4 |
| **Predator cue** | **Absence** | / | / | / | 11.2e-3 ± 6.1e-4 |
|  | **Presence** | / | / | / | 9.1e-3 ± 6.2e-4 |

| Analysis 2 |  | Entrance into the final instar F-0 | | | Predator cue treatment |
| --- | --- | --- | --- | --- | --- |
|  |  | Dev. time (days) | Mass (mg) | Growth rate (ln) | Growth rate (ln) |
| **Sex** | **Male** | 173 ± 0.63 | 28.5 ± 0.26 | 19.4e-3 ± 8.1e-5 | 9.5e-3 ± 5.9e-4 |
|  | **Female** | 176 ± 0.67 | 30.8 ± 0.28 | 19.5e-3 ± 8.7e-5 | 7.8e-3 ± 5.5e-4 |
| **Temperature** | **20 ˚C** | 181 ± 0.72 | 32.7 ± 0.28 | 19.3e-3 ± 10.7e-5 | 8.7e-3 ± 7.4e-4 |
|  | **24 ˚C** | 172 ± 0.71 | 29.7 ± 0.27 | 19.8e-3 ± 10.4e-5 | 8.9e-3 ± 7.2e-4 |
|  | **28 ˚C** | 171 ± 0.63 | 27.0 ± 0.25 | 19.3e-3 ± 9.4e-5 | 8.5e-3 ± 6.5e-4 |
| **Urbanization type** | **Rural** | 173 ± 0.65 | 29.8 ± 0.28 | 19.6e-3 ± 8.3e-5 | 8.6e-3 ± 5.7e-4 |
|  | **Urban** | 175 ± 0.66 | 29.3 ± 0.28 | 19.3e-3 ± 8.4e-5 | 8.7e-3 ± 5.8e-4 |
| **Predator cue** | **Absence** | / | / | / | 8.8e-3 ± 5.7e-4 |
|  | **Presence** | / | / | / | 8.6e-3 ± 5.8e-4 |
