# Supplementary figures and images for: Latitude‐specific urbanization effects on life history traits in the damselfly Ischnura elegans
Source: Evol Appl. 2023 Aug 1;16(8):1503–15. doi: 10.1111/eva.13583 (PMC10445092; doi:10.1111/eva.13583)

FLake model

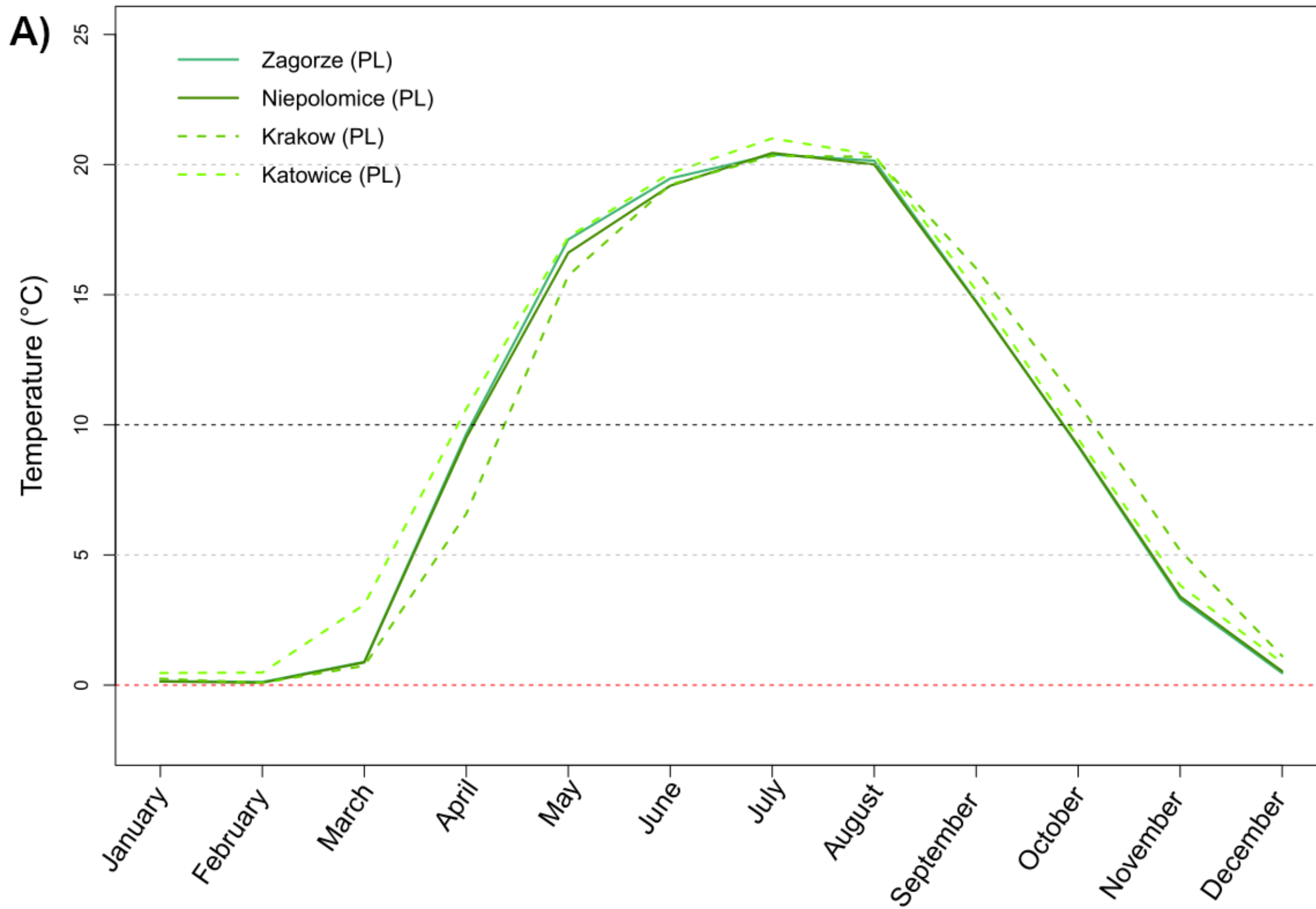

FLake model

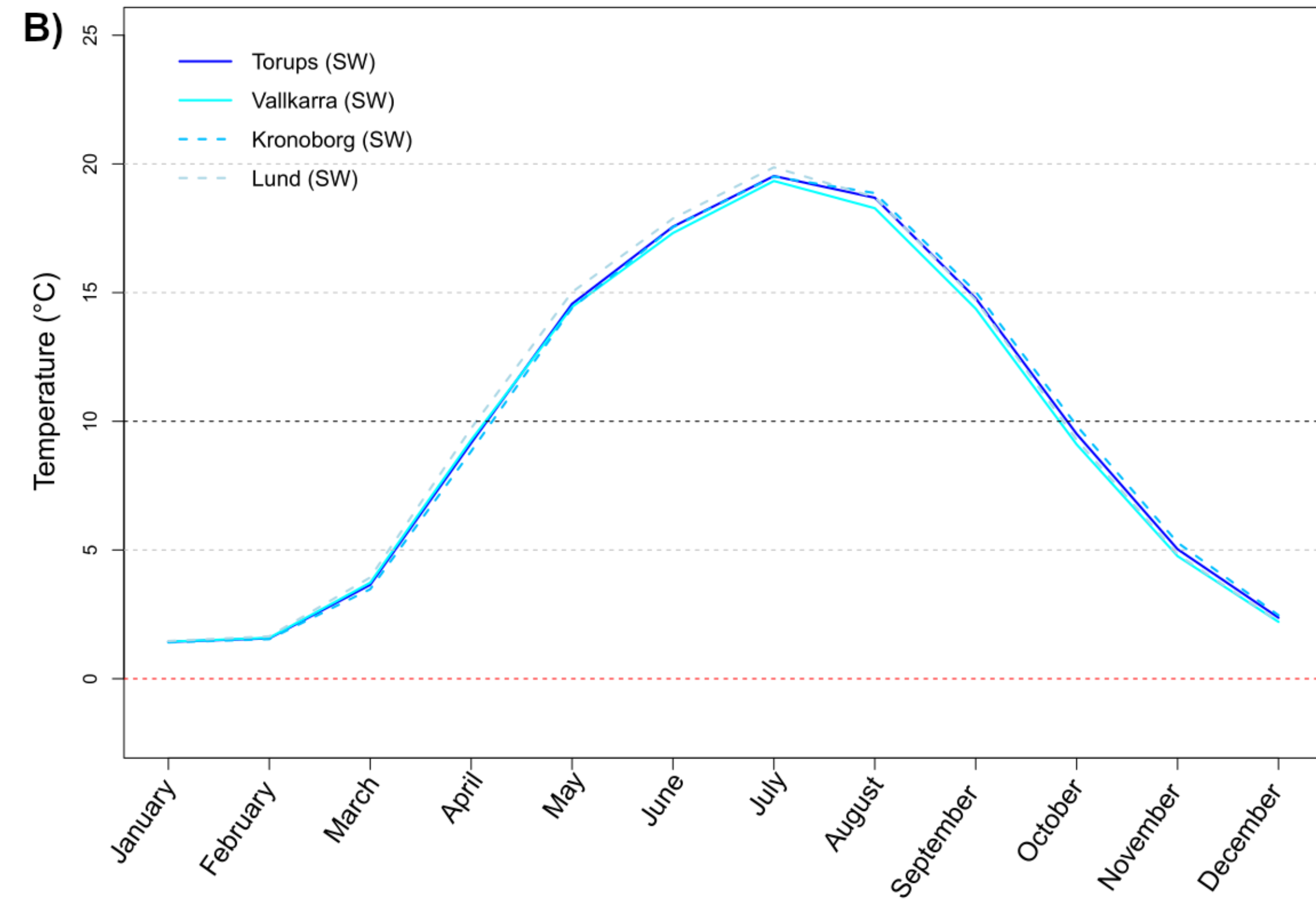

Logger

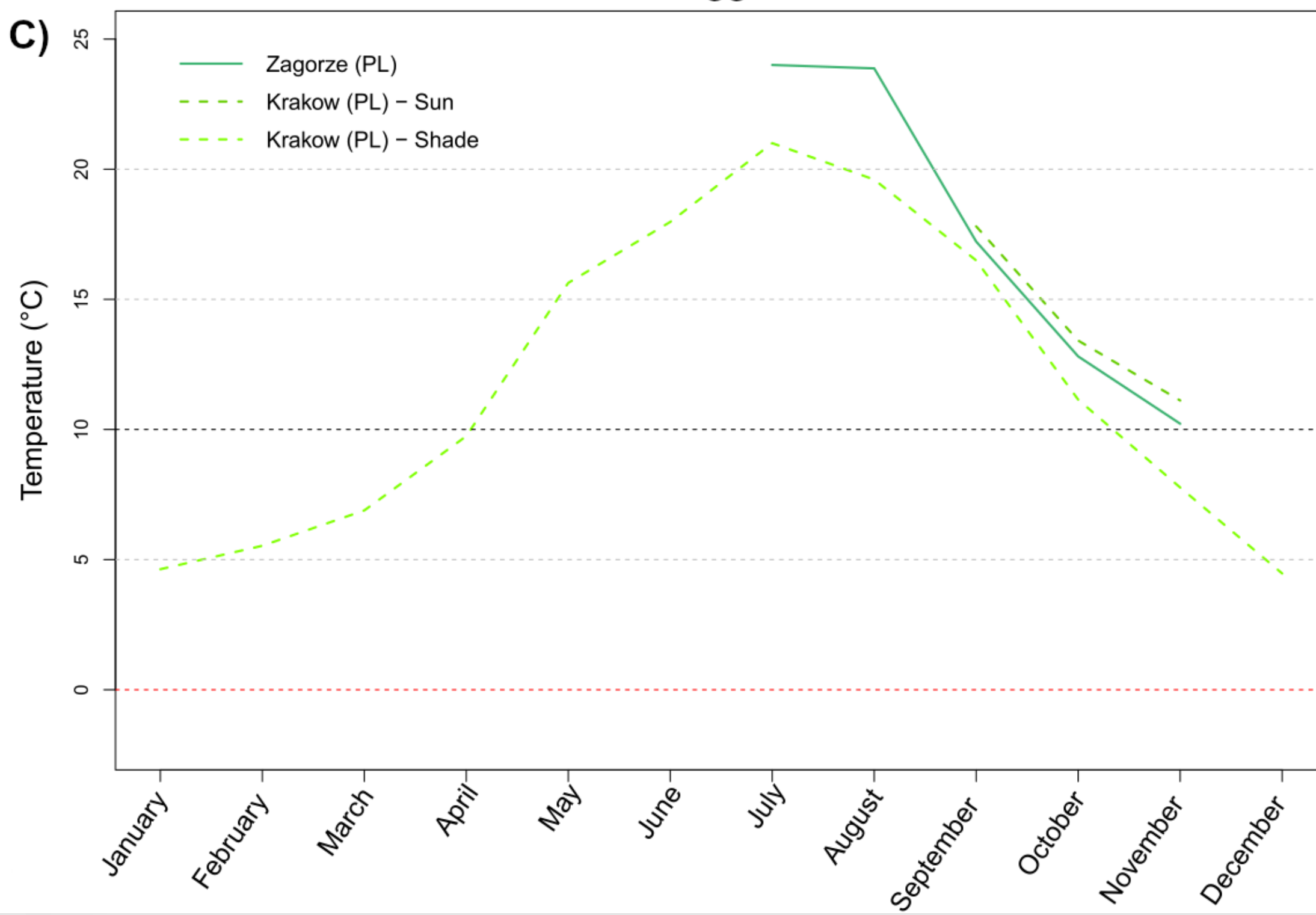

Logger

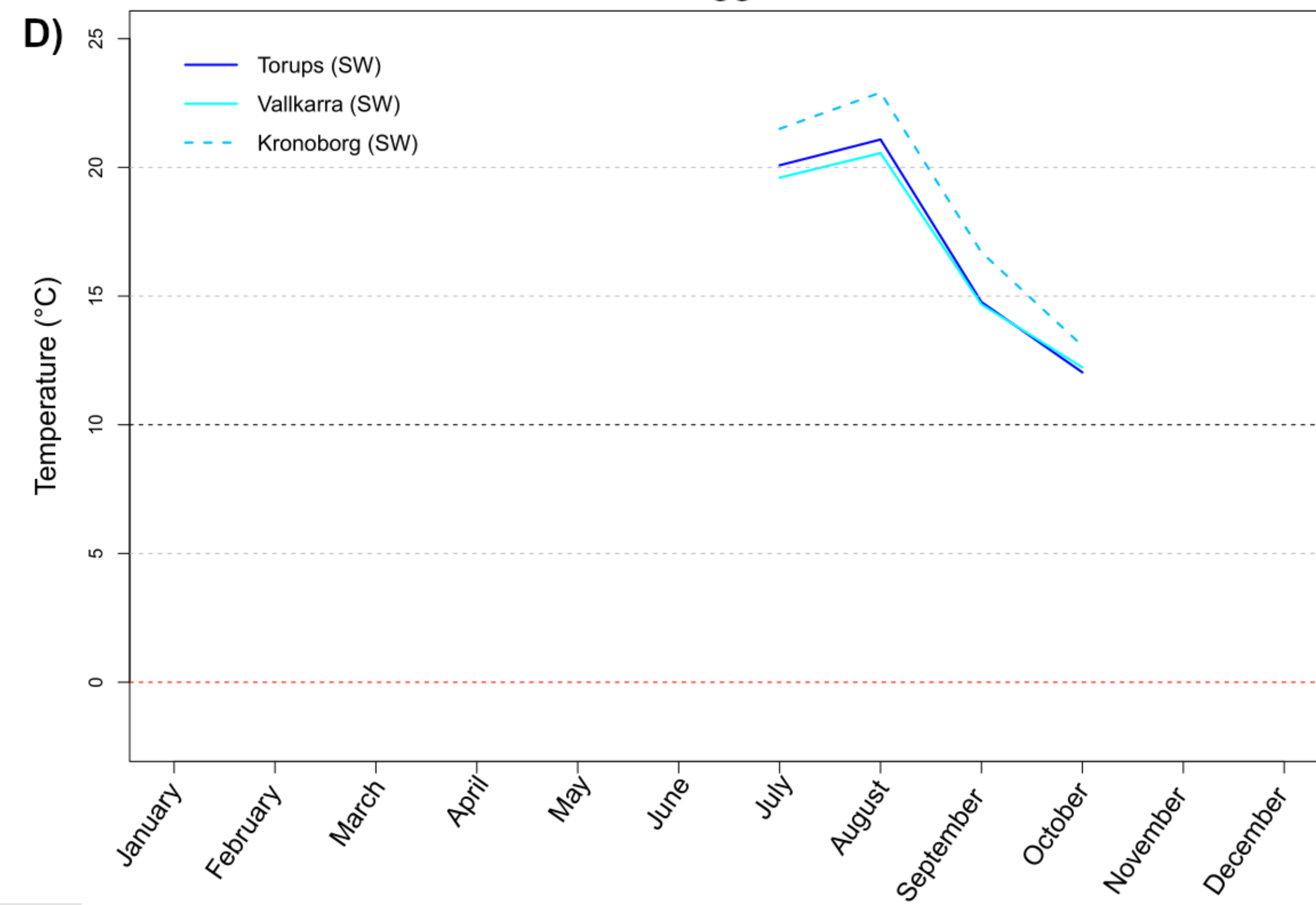

Supplement: Supplementary file 2 — Figure S1 [file EVA-16-1503-s003.pdf]

Central latitude

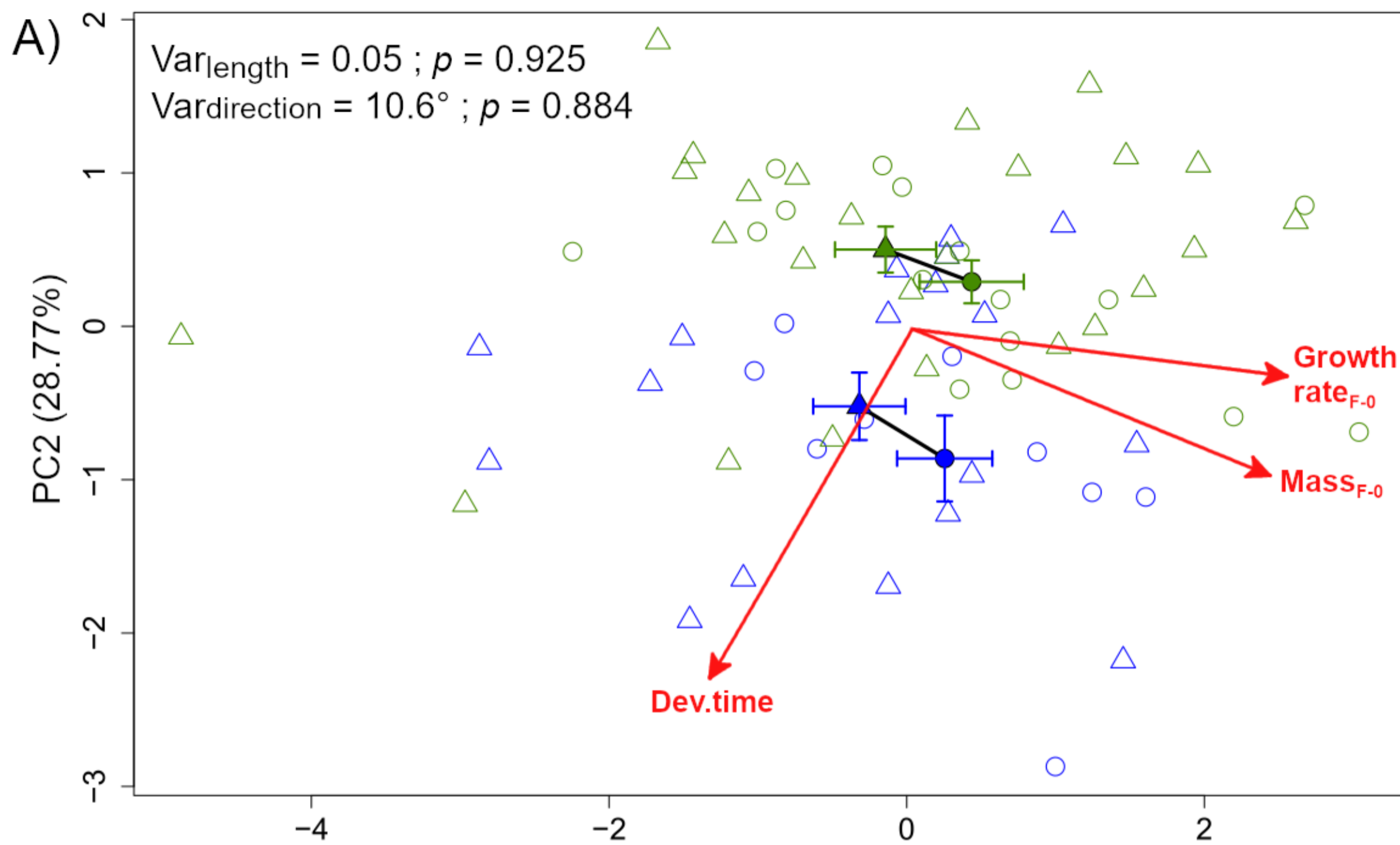

High latitude

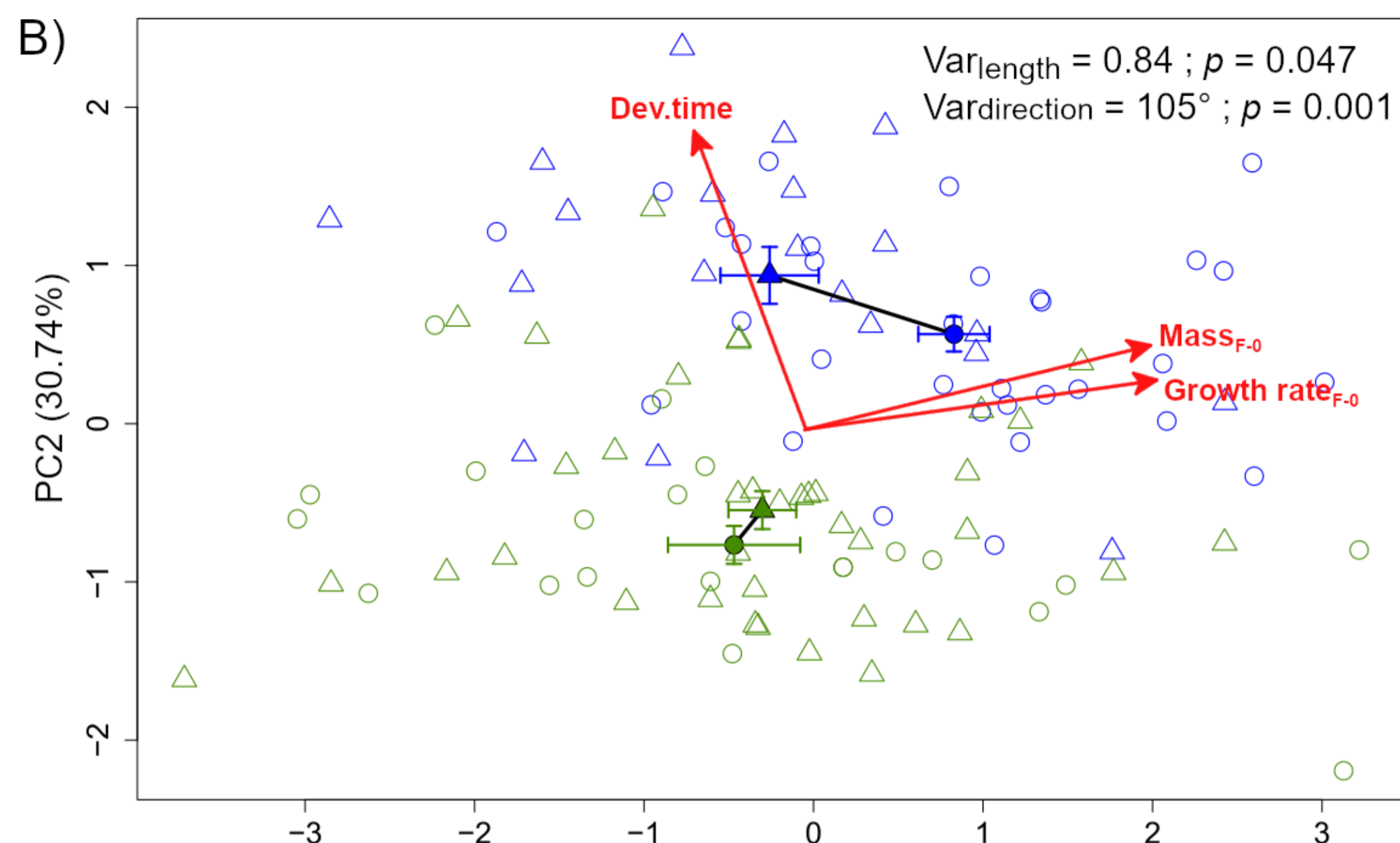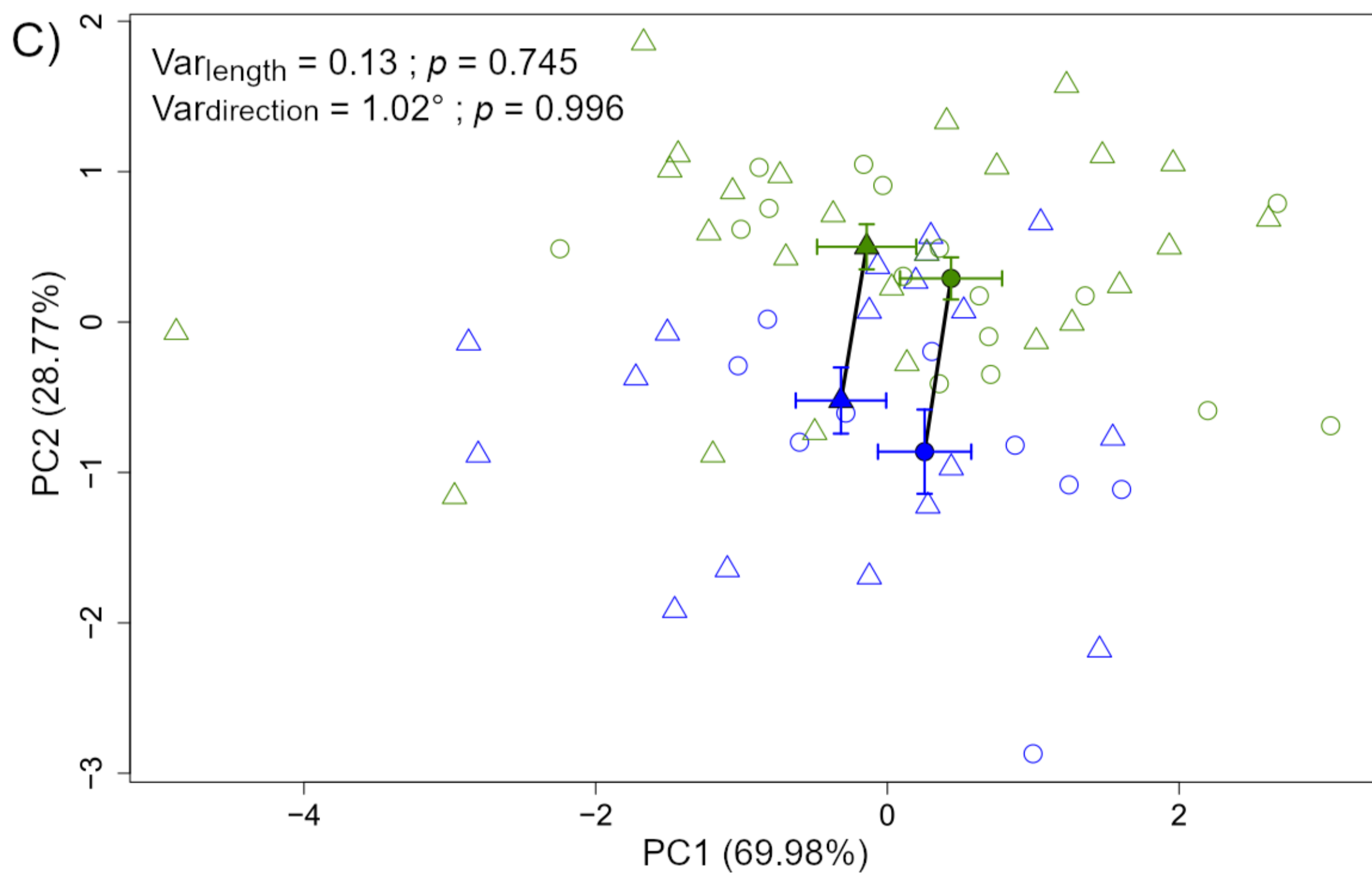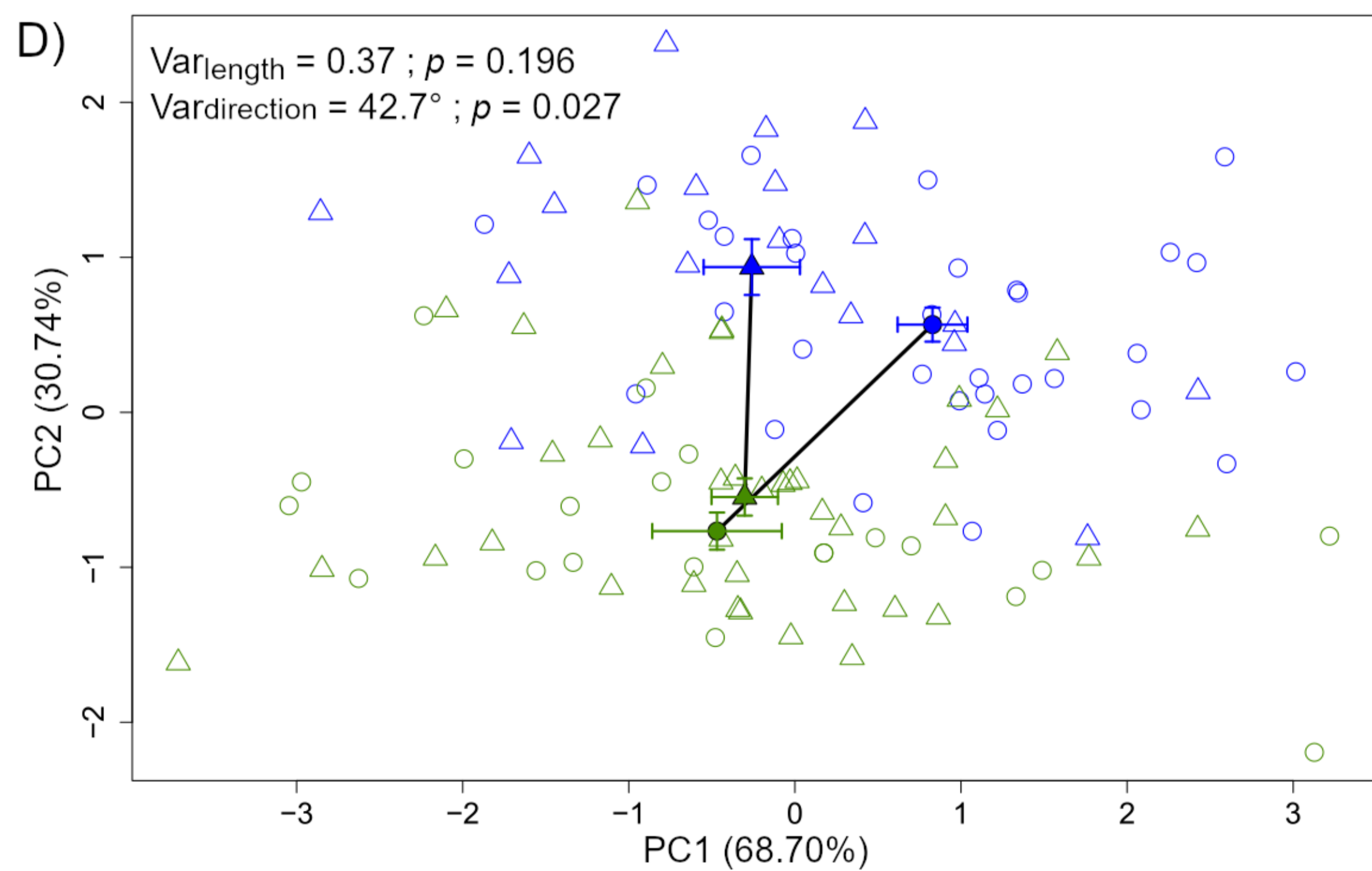

○ Rural 20 °C    ○ Rural 24 °C  
△ Urban 20 °C    △ Urban 24 °C

Supplement: Supplementary file 3 — Figure S7 [file EVA-16-1503-s001.pdf]

# Central latitude

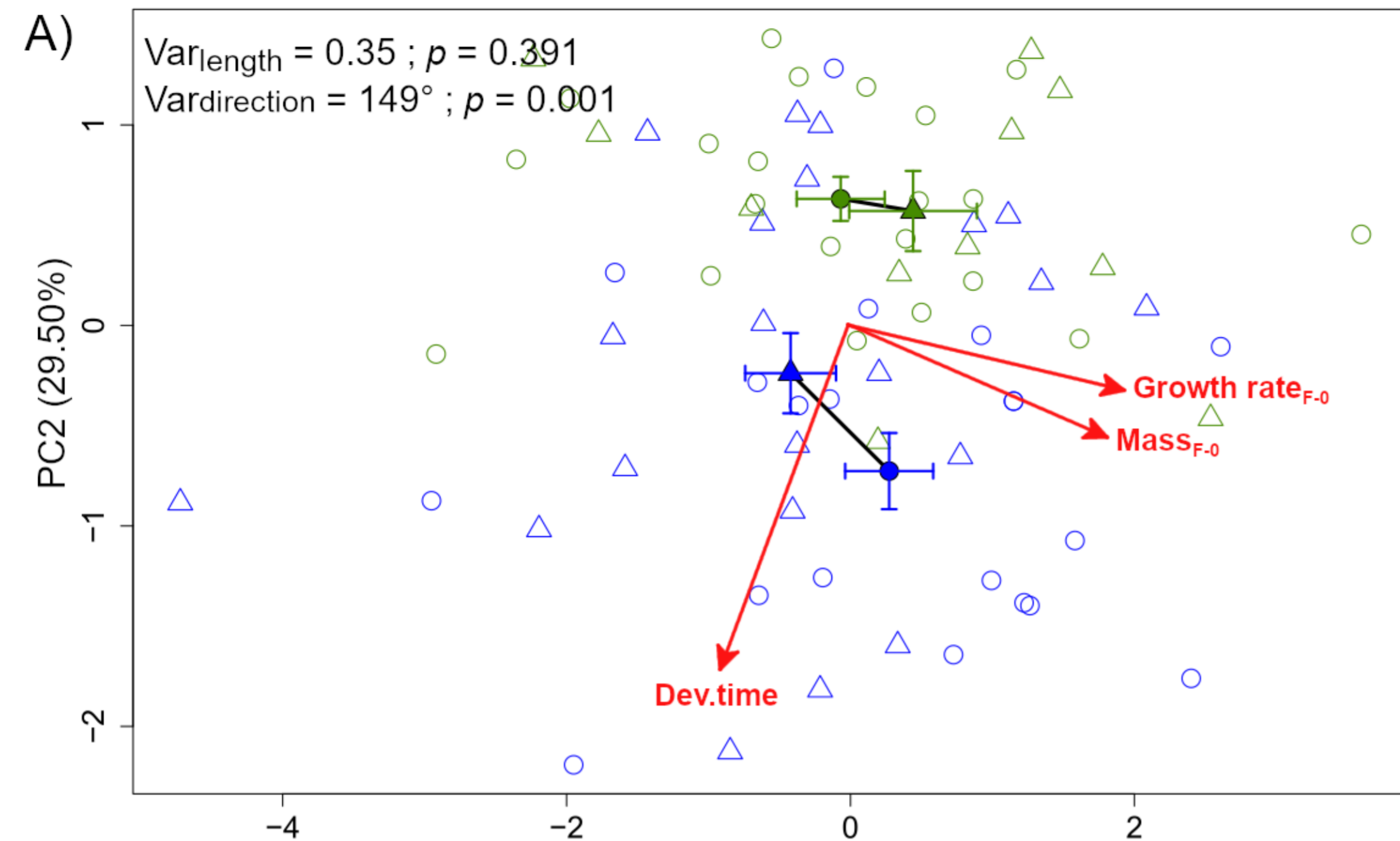

# High latitude

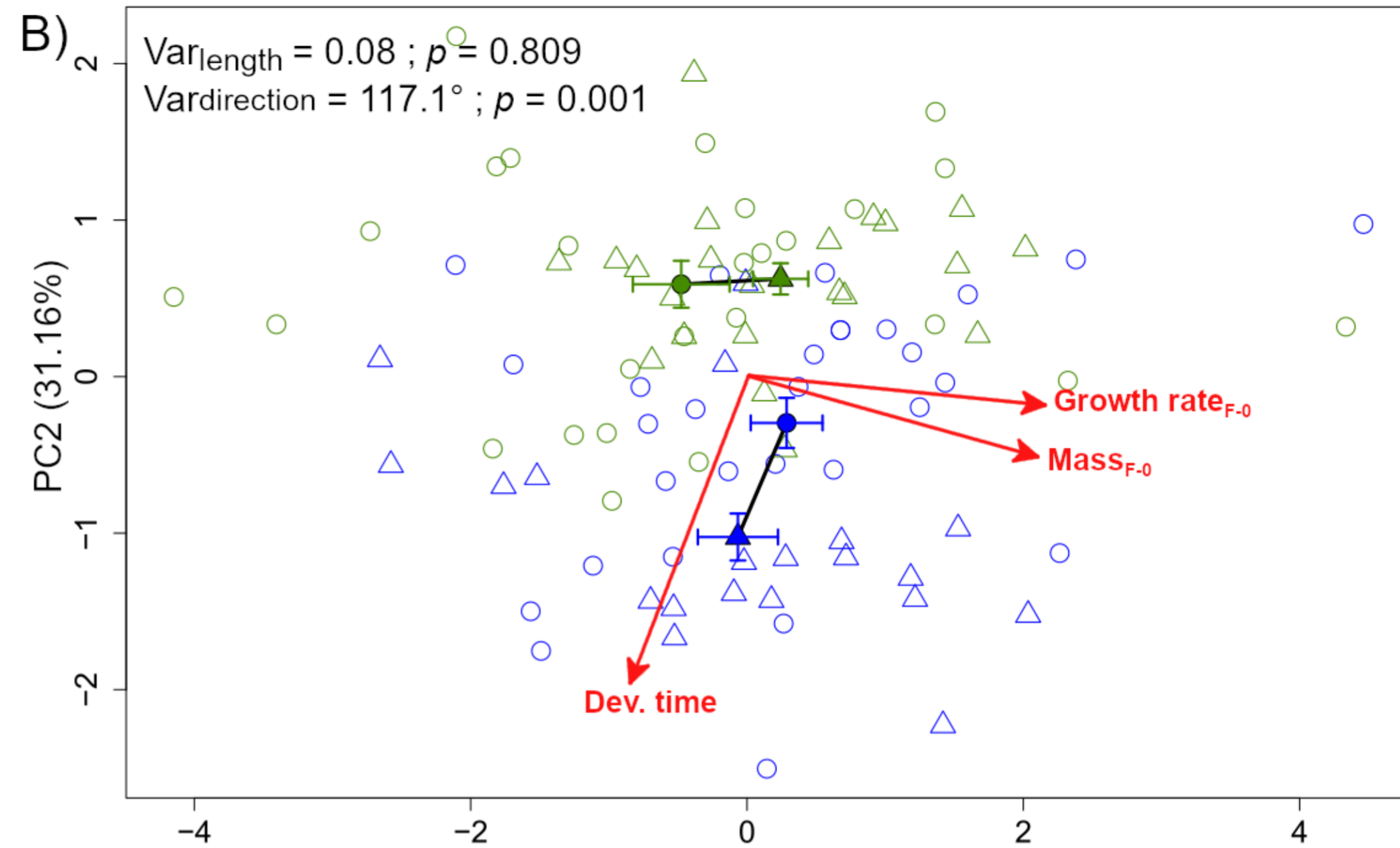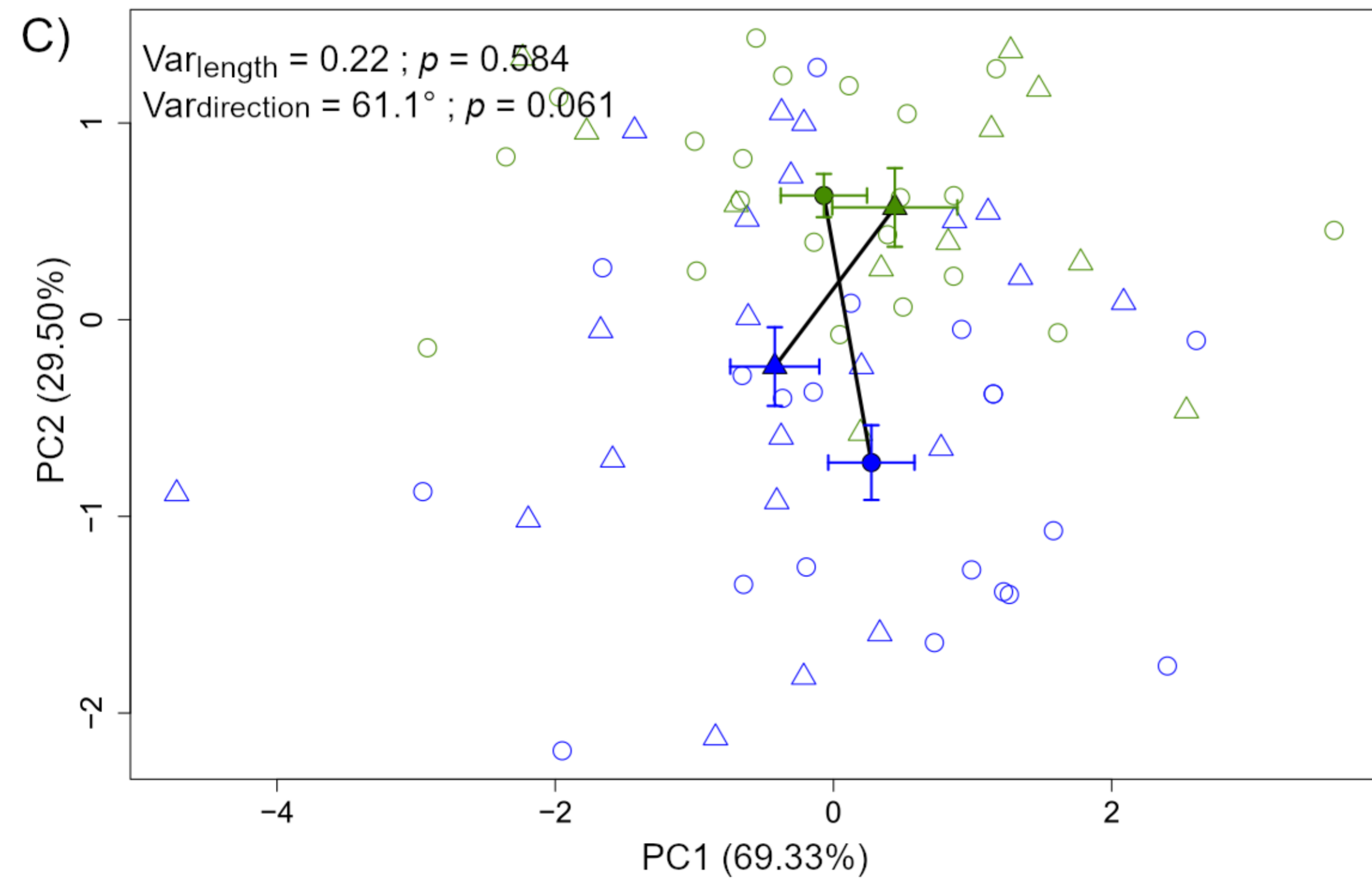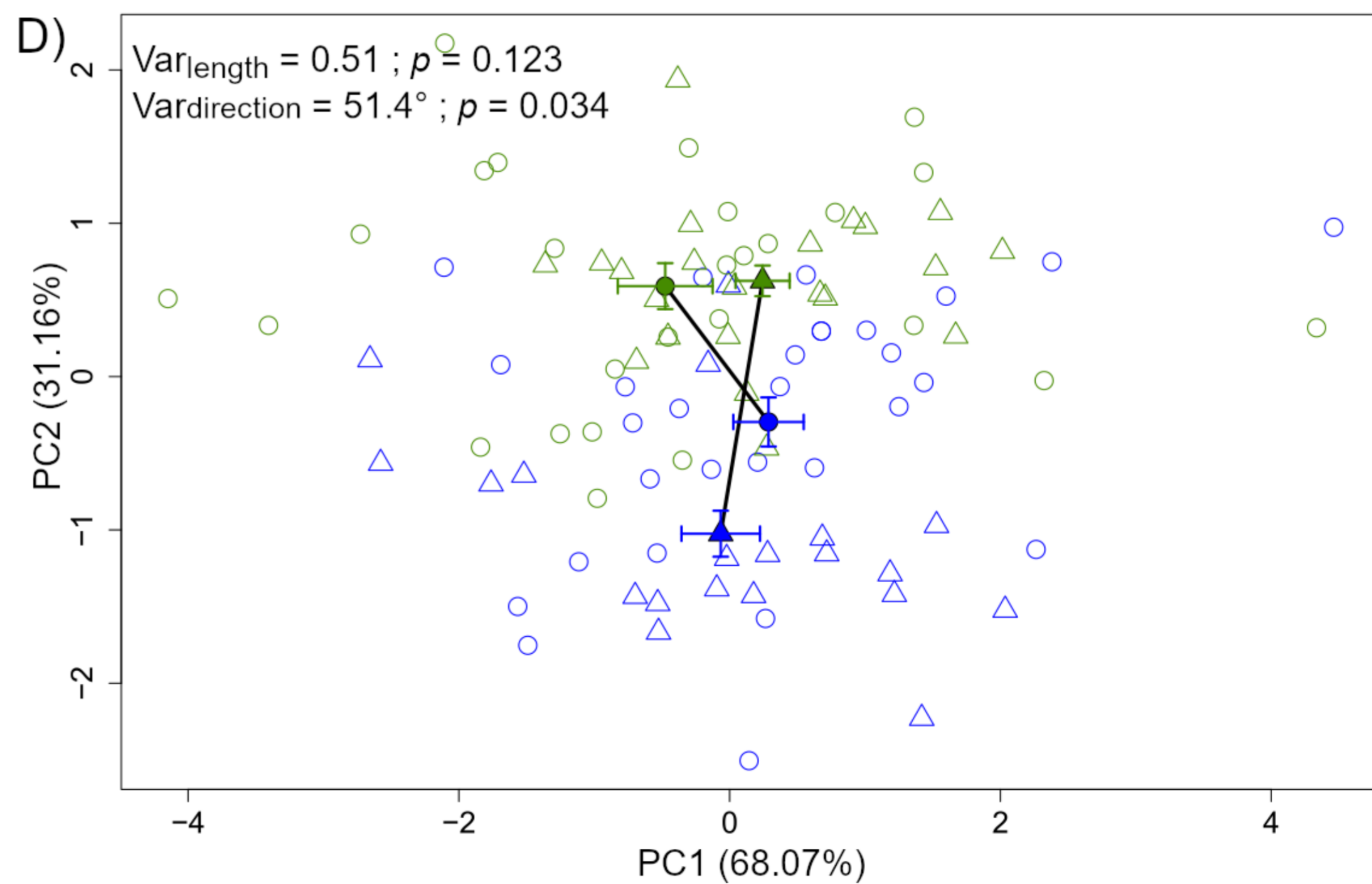

○ Rural 20 °C    ○ Rural 24 °C  
 △ Urban 20 °C    △ Urban 24 °C

Supplement: Supplementary file 4 — Figure S8 [file EVA-16-1503-s006.pdf]

Males

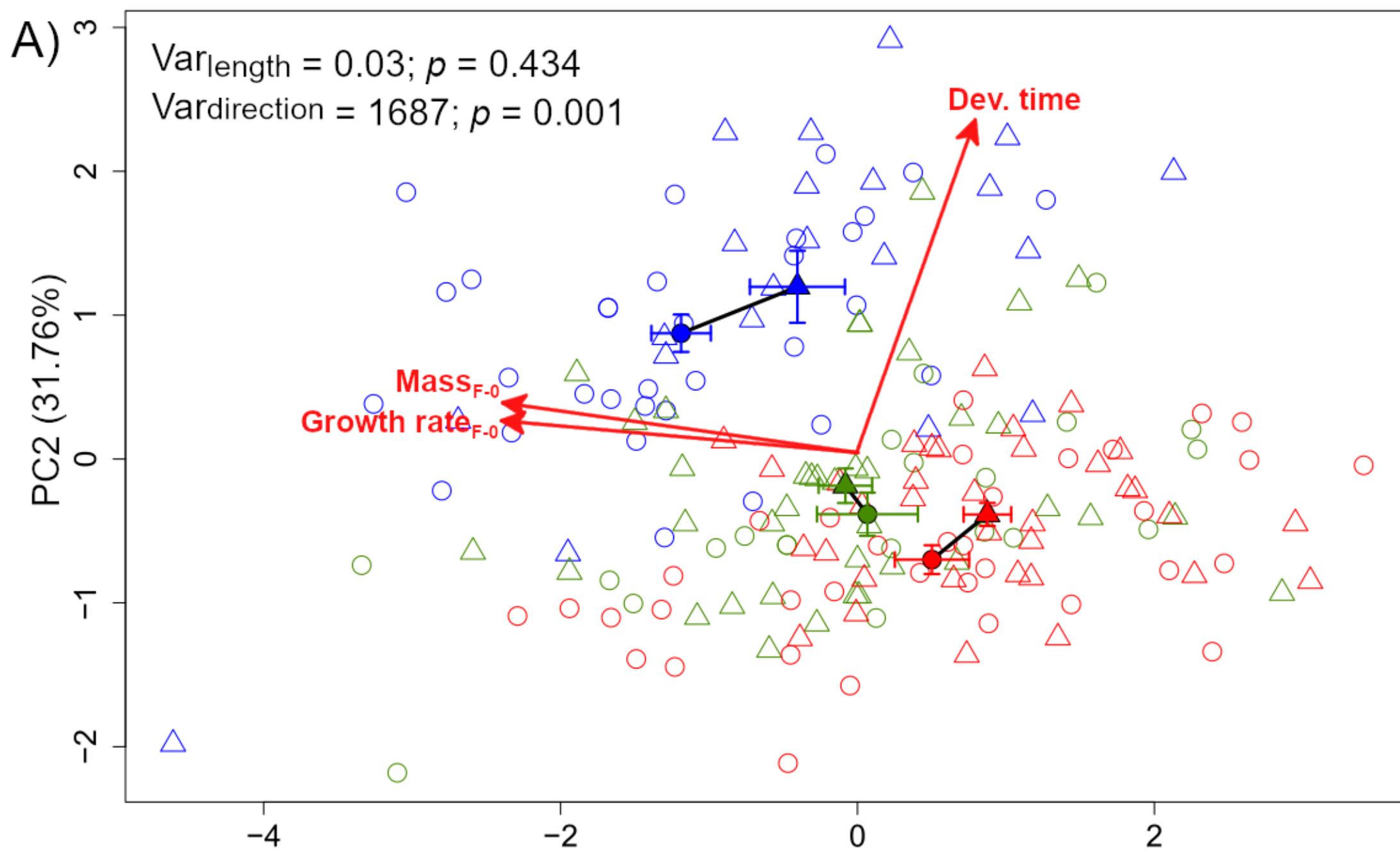

Females

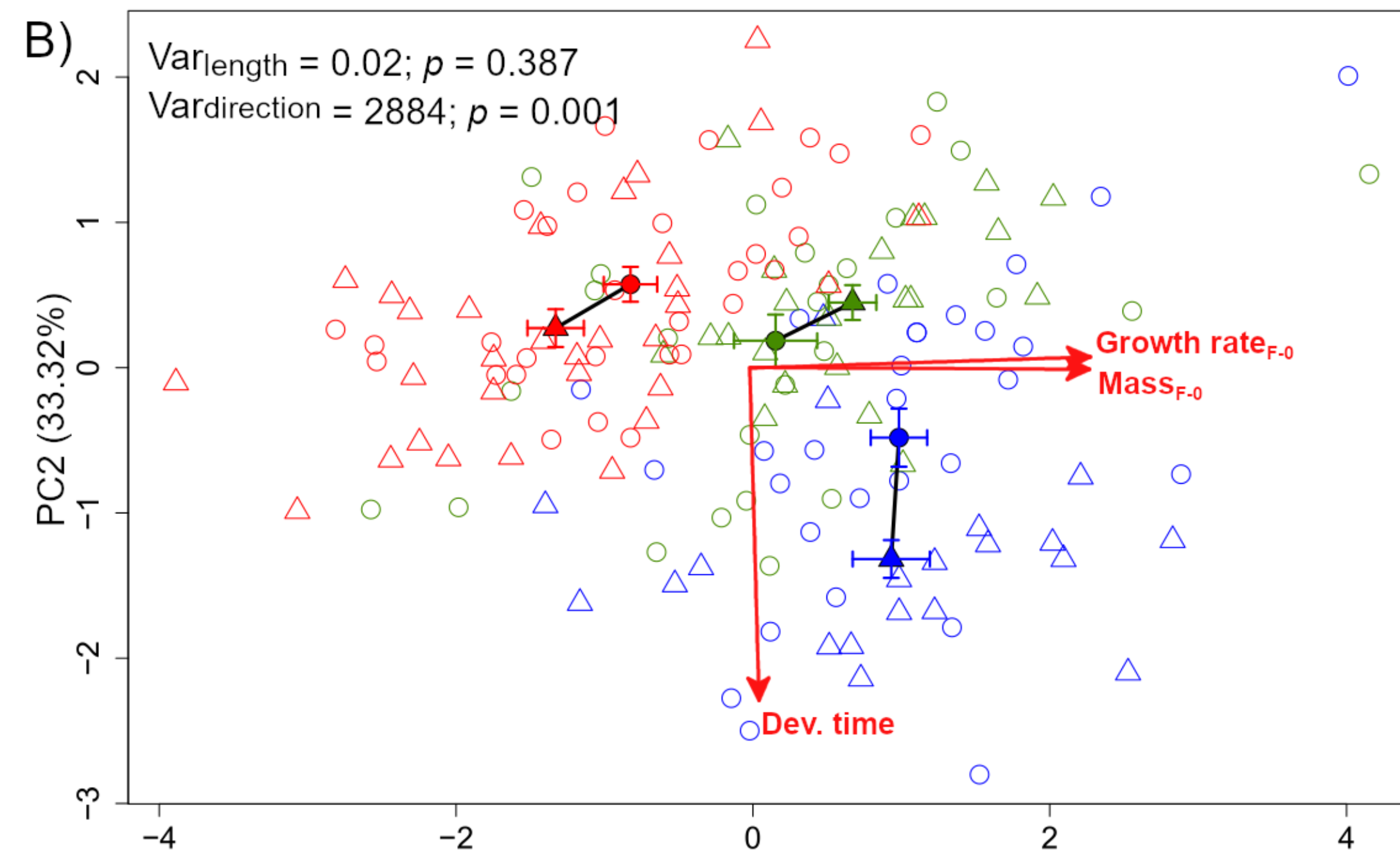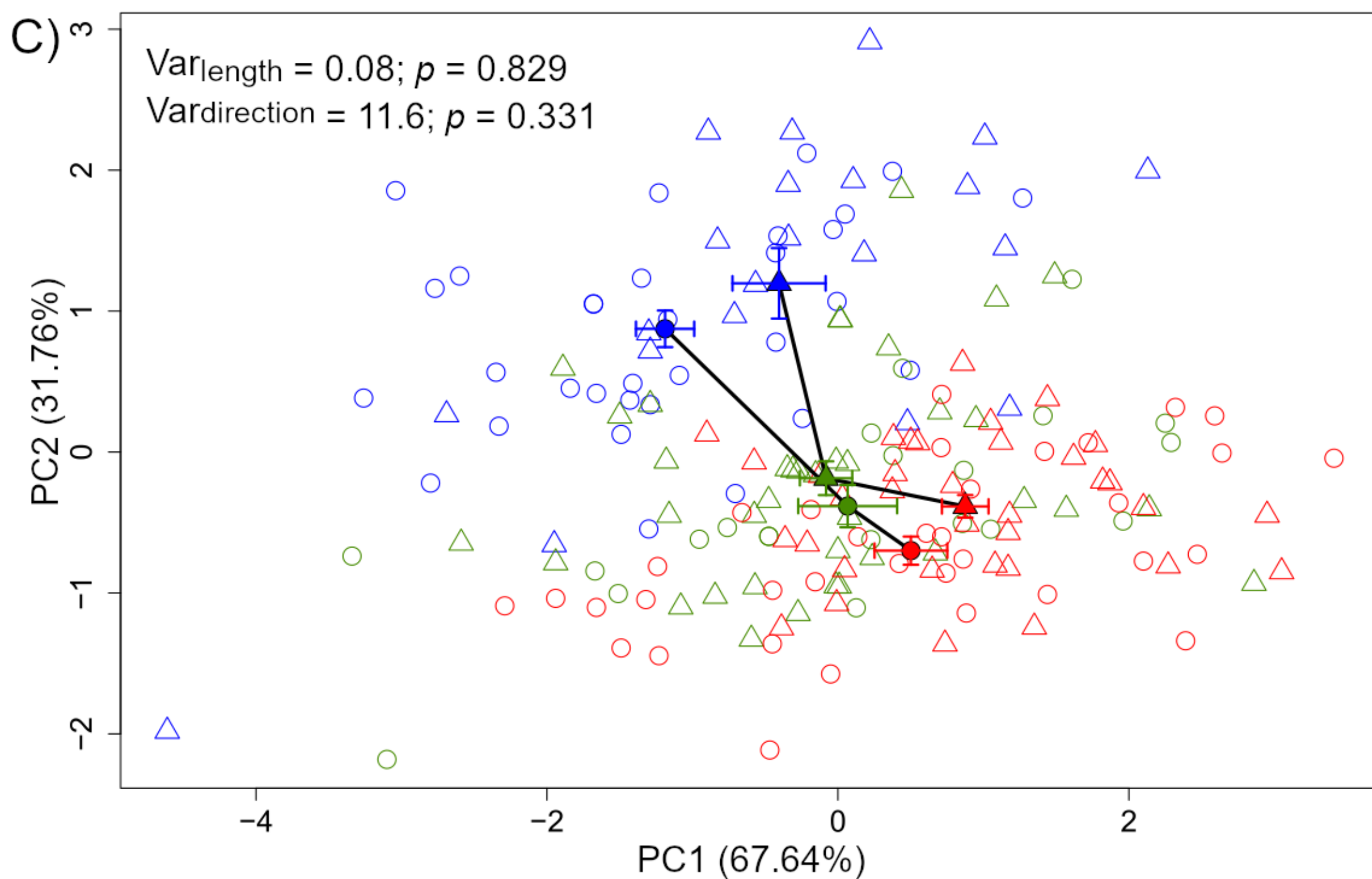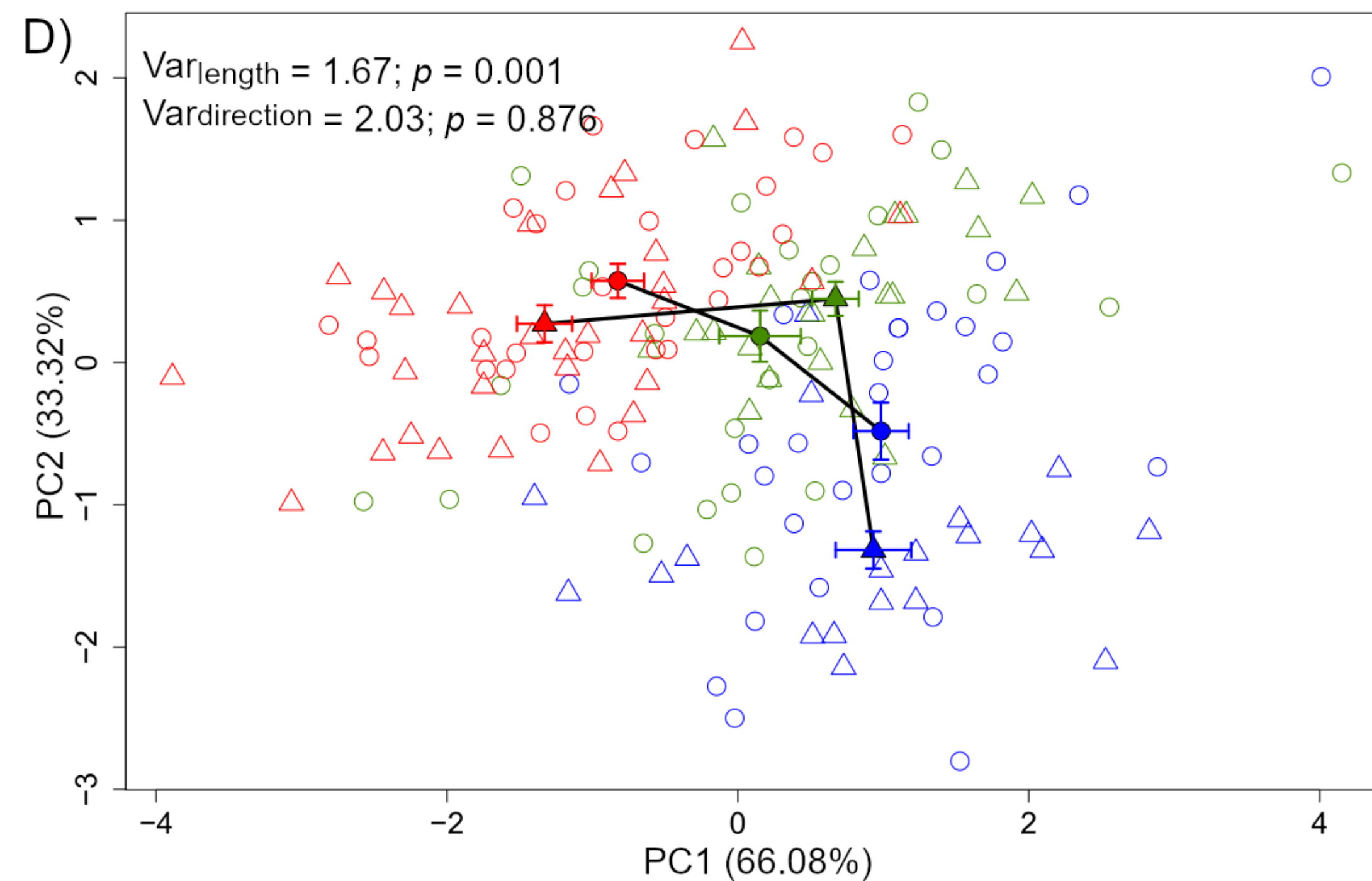

○ Rural 20 °C   ○ Rural 24 °C   ○ Rural 28 °C  
△ Urban 20 °C   △ Urban 24 °C   △ Urban 28 °C

Supplement: Supplementary file 5 — Figure S10 [file EVA-16-1503-s002.pdf]
